# Comprehensive Map of Molecules Implicated in Obesity

**DOI:** 10.1371/journal.pone.0146759

**Published:** 2016-02-17

**Authors:** Jaisri Jagannadham, Hitesh Kumar Jaiswal, Stuti Agrawal, Kamal Rawal

**Affiliations:** Department of Biotechnology, Jaypee Institute of Information Technology, Noida [UP]-201 307, India; National Research Council of Italy (CNR), ITALY

## Abstract

Obesity is a global epidemic affecting over 1.5 billion people and is one of the risk factors for several diseases such as type 2 diabetes mellitus and hypertension. We have constructed a comprehensive map of the molecules reported to be implicated in obesity. A deep curation strategy was complemented by a novel semi-automated text mining system in order to screen 1,000 full-length research articles and over 90,000 abstracts that are relevant to obesity. We obtain a scale free network of 804 nodes and 971 edges, composed of 510 proteins, 115 genes, 62 complexes, 23 RNA molecules, 83 simple molecules, 3 phenotype and 3 drugs in “bow-tie” architecture. We classify this network into 5 modules and identify new links between the recently discovered fat mass and obesity associated FTO gene with well studied examples such as insulin and leptin. We further built an automated docking pipeline to dock orlistat as well as other drugs against the 24,000 proteins in the human structural proteome to explain the therapeutics and side effects at a network level. Based upon our experiments, we propose that therapeutic effect comes through the binding of one drug with several molecules in target network, and the binding propensity is both statistically significant and different in comparison with any other part of human structural proteome.

## Introduction

Obesity, a complex condition with serious medical, psychological and social consequences, affects millions of people across the world [1]. In addition, rising numbers of juvenile onset obesity cases contribute to increased incidence of time-dependent complications of obesity such as insulin resistance, non-insulin-dependent diabetes mellitus, hypertension, coronary artery disease and other cardiac disorders often grouped as "metabolic syndrome X" [2–3]. The pathophysiology of obesity is influenced by several factors such as candidate genes and their expression, single nucleotide polymorphisms, proteins, metabolic pathways and their perturbations due to mutations, nutrition, exercise, gut microbes, and diseases, e.g. hypothyroidism [4–5]. Experts recommend that increase in physical activity and reduction in intake of high calorie foods, can act as possible deterrent to obesity epidemic. Numerous studies have examined the use of medicines [6–7] and surgery [8] as possible treatment measures, although clinical studies also indicate that recurrences are high in people who have lost weight through diet, exercise or medication [9].

Adipose tissue is central to the regulation of energy balance. In the pathophysiology of obesity, chronic adipose tissue inflammation is a hallmark [10]. Two functionally different types of fats are present in mammals: white and brown adipose tissue. White adipose tissue is the primary site of triglyceride storage whereas brown adipose tissue is implicated in energy expenditure. The latter has an ability to counteract obesity [11]. Adipogenesis, namely the formation of adipose tissue begins with the commitment of mesenchymal stem cells (MSCs) to the adipocyte lineage, followed by terminal differentiation of preadipocytes to mature adipocytes [12]. The adipose tissue storage is influenced by environmental and genetic factors. The environmental influence generally depends on the individual’s life-style, for instance, food intake and physical activity. The importance of genetic factors in obesity has been demonstrated by twin studies, adoption studies and segregation analyses [13–14].

The literature data pertaining to obesity is vast and complex. For instance, ‘obesity’ as a keyword in PubMed yielded over 13,000 results (hits) for a single year 2011. It was increased by 11,612 hits in 2012 and 11,177 hits in 2013, showing that literature data is growing at rapid pace. In addition to proteins and other molecules, these abstracts contain reports from clinical, genetic, mutational and meta-studies. To construct comprehensive molecular map of molecules reported in obesity, we used information from full length articles using deep curation model [15]. A deep curation model performs better than text mining methods, particularly in terms of accuracy, but has the disadvantage of being labour intensive and time consuming [16]. Given this constraint, it is difficult to curate large number of papers published every year and such a resource will become obsolete in absence of regular revision and updates by experts. Therefore, we decided to develop a hybrid system combining text mining systems and deep curation strategy to screen large amount of published data available on obesity to provide up-to-date information.

Networks pervade our lives as exemplified by worldwide webs, internet, small world networks, electricity grids, social networks, topology of food webs, citation networks as well as metabolic networks. To understand the role of networks in complex diseases, there were several attempts to construct disease networks [17–19]. A research group built a pathway on an autoimmune disease, “Rheumatoid arthritis” using microarray data [20]. In biological systems, at molecular or cellular levels, several reconstructions of comprehensive pathways have been conducted using published literature data. These include compiling human cell-cycle events by Kohn [21], comprehensive maps of EGFR pathway [22], Toll-like receptor signalling pathway [23] and RB/E2F pathway [24]. Apart from these, researchers have also used microarray data [25], protein-protein interaction data [26–27], co-cited data [28] as well as literature data [29] to construct networks. Despite all these efforts, there is plenty of scope to expand the role of networks in disease pathophysiology.

Human Obesity Gene Map 2005 is considered to be one of the best information resource for genes implicated in obesity [30]. The Human Obesity Gene Map 2005 provides evidence from single-gene mutation obesity cases, Mendelian disorders exhibiting obesity as a clinical feature, transgenic and knockout murine models relevant to obesity, quantitative trait loci (QTL) from animal cross-breeding experiments, association studies with candidate genes, and linkages from genome scans and genes or markers that have been shown to be associated or linked with obesity phenotype. We identified 379 genes reported in obesity from Human Obesity Gene Map and included in our proposed network. Transcription factors play an important role in conversion of pre-adipocytes to adipocytes and involved in several other mechanisms pertaining to obesity pathophysiology. For this reason, we retrieved 114 transcription factors from DGAP (Diabetes Genome Anatomy Project) & GenMapp (http://www.genmapp.org/default.html). Apart from these, we identified an independent set of 33 genes reported in obesity literature (See Table A in S2 File for list of molecules & Table B in S2 File for experimental evidence). This work was complemented by mining over 35,000 genes in 96,219 abstracts using perl scripts. Through text-mining, we found 4,274 genes as first round of ‘hits’ (See Methods). Since text mining systems are known to produce large number of false hits, therefore we screened these hits manually and removed gene names matching with common English words, abbreviations and methodology terms using various types of filters (See examples provided on our website http://tinyurl.com/d74r9xy as well as in S3 File). Out of 4,274 hits, we label 1,268 genes as positive hits and 3,006 as false positive hits (See Table C in S2 File). Text mining system also reported several recently published molecules such as fat mass and obesity associated (FTO) and omega-3 fatty acid receptor 1 (GPR120) [31–32].

**Fig 1:**
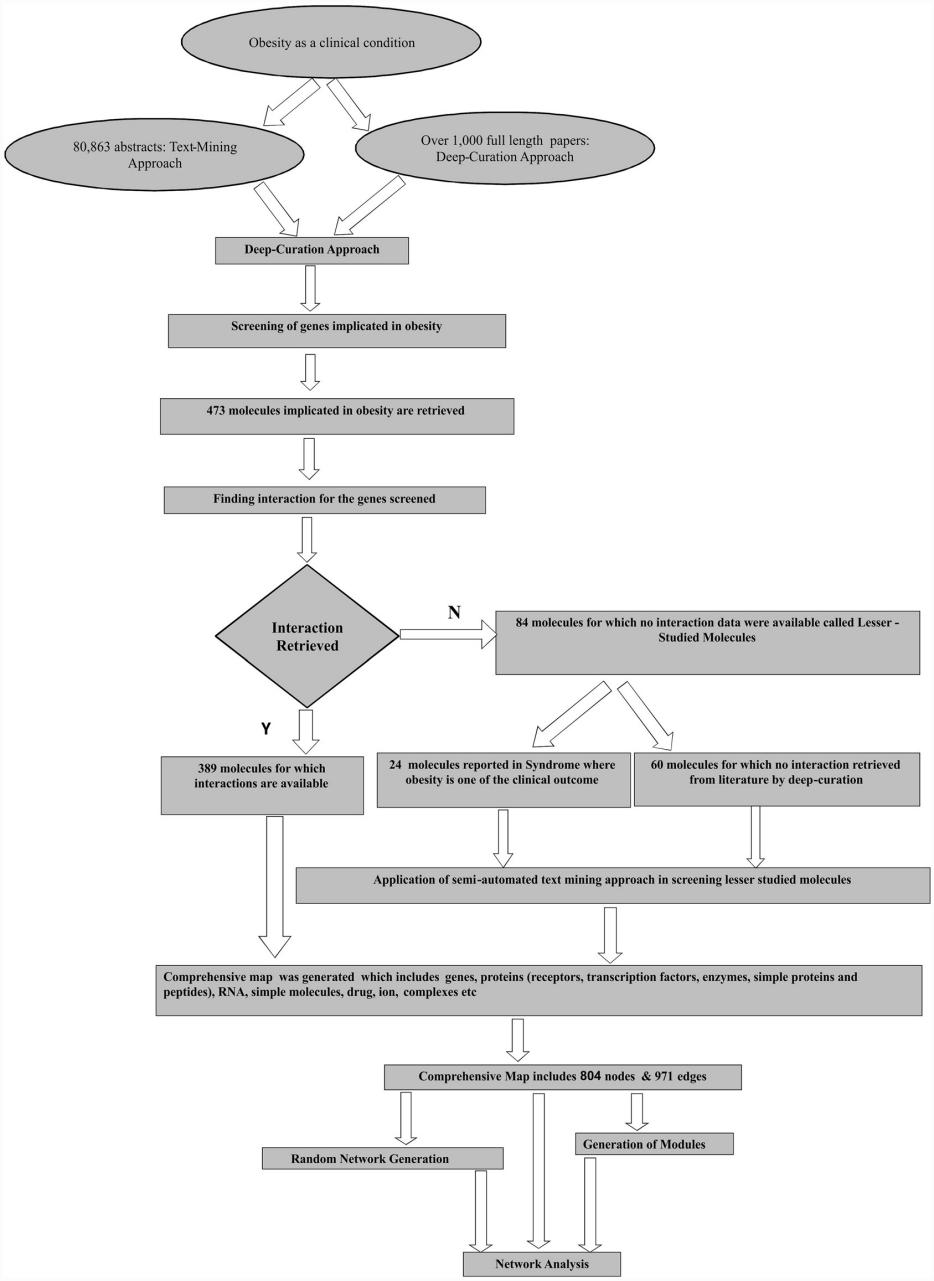
Flowchart for generation of comprehensive map.

Based upon these techniques, we constructed two datasets (A and B) to create comprehensive network. Set A consist of 473 genes and proteins retrieved through deep curation strategy whereas set B consist of 1268 genes retrieved through semi-automatic text mining system. We started with this set of molecules as a 'partial list' of the proposed comprehensive network and expanded by adding more molecules based upon interactions reported in literature in context of obesity. The final comprehensive map was constructed based upon genes, proteins, receptors, transcription factors, enzymes, ion channels, drugs, RNA molecules, simple molecules and their relationships (See Fig 1).

## Results

### General features of the map

We screened over one thousand research articles manually and more than 96,219 abstracts (published till December 2012) using text mining system. The majority of molecules identified in this study can be tracked to sources such as human obesity gene map database update 2005, GenMapp and miscellaneous literature reports (Table A in S2 File). We have prepared a resource base where each molecule is linked with its research article. Each paper is curated manually and the portion of text denoting gene (molecule) or its interaction with other molecules in context of obesity is highlighted. The information on interactions of molecule is given on our website (Supplementary Folder 1: Gene interaction evidence: “http://tinyurl.com/nc3yjj7” & A in S3 File & Table D in S2 File). During this study, we encountered set of molecules which are found to be involved in syndromes where obesity is one of the clinical outcomes. Since the direct evidence on the role of these molecules in obesity is not known, we decided to include them as an independent part of the proposed map. We label this set as ‘lesser studied (reported) group’ due to paucity of literature data. To illustrate, ALMS1 gene is related with an “Alstrom syndrome 1” where obesity is a frequent clinical outcome in patients [33], but, direct evidence of linking ALMS1 with obesity is not reported in literature. Similarly, Gamma-aminobutyric acid A receptor gamma 3 (GABRG3) is an early childhood obesity gene reported in Prader-Willi syndrome [34], but, direct experimental evidence is not known. Likewise, genes reported from X-linked mutation studies and linkage studies could not be placed in the main network due to sparse experimental or interaction data. Therefore, out of 473 molecules, we included 389 molecules in the proposed network and the rest 84 molecules were reported as an independent set (See Table E in S2 File). The process, of incorporation of lesser studied molecules in proposed network, is elaborated in the following sections.

**Fig 2:**
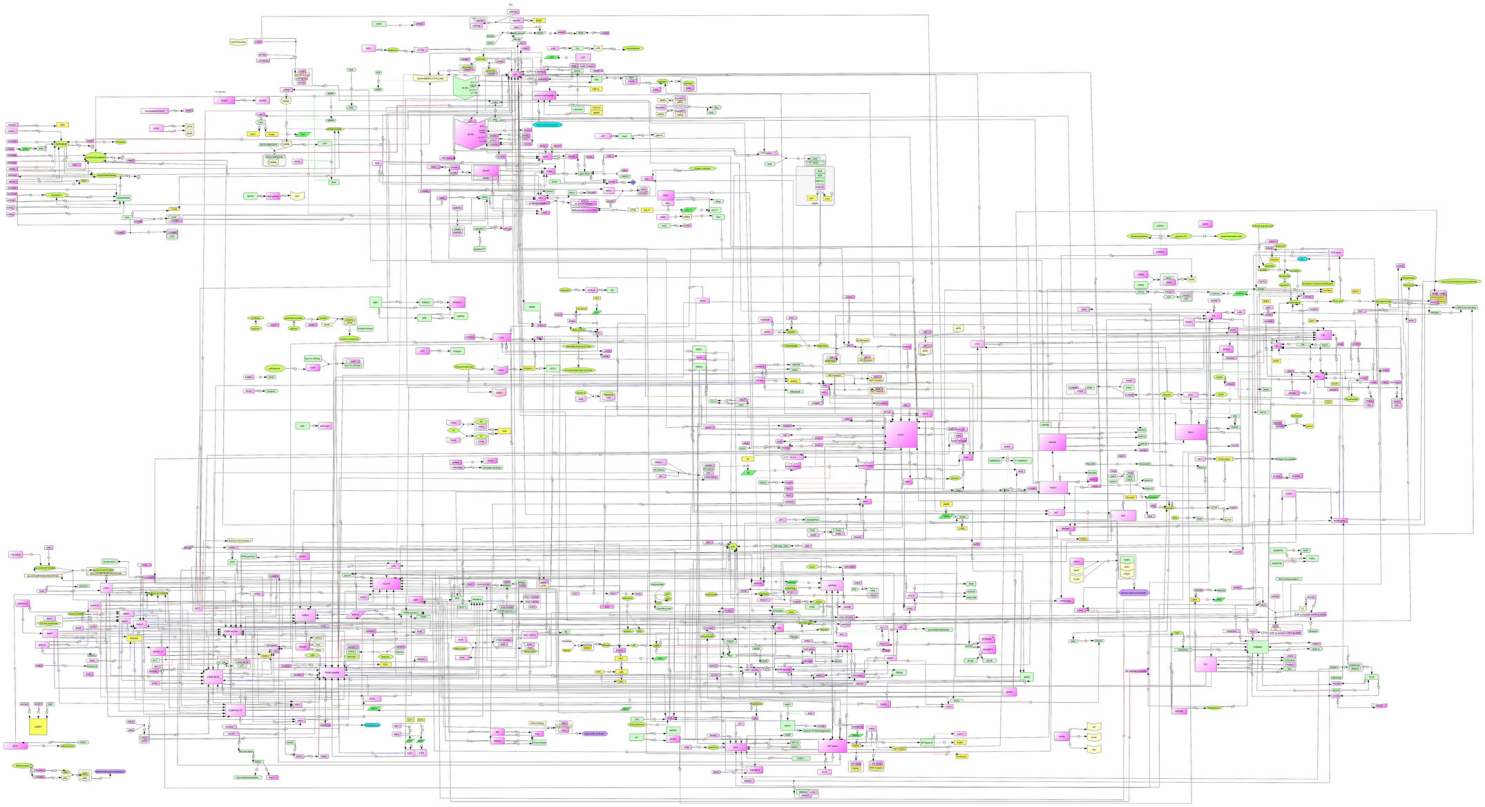
A comprehensive map of obesity in human. (Also see URL:http://tinyurl.com/dykn8fd).

In Fig 2, we show comprehensive map of molecules that was manually assembled based on the published literature. Various entities of the network, e.g. genes, proteins and their modifications, protein complexes are described using standard Systems Biology Markup Language (SBML) with the help of Cell Designer 4.1 software[35] and Systems Biology Graphical Notation (SBGN)[36](www.sbgn.org). The nodes (also known as species) represent molecules that participate in a given reaction. The edges represent reactions among nodes. The resulting network on obesity consists of 804 nodes (includes set A molecules as well as other genes/molecules interacting with set A) and 971 edges. These 804 nodes are categorized as 510 proteins, 115 genes, 1 ion, 3 drugs, 3 degraded molecules, 62 complexes, 23 RNA molecules, 83 simple molecules, 3 phenotypes and 1 unknown molecule (See Table F in S2 File). The reactions are categorised as follows: 848 state transitions, 33 transcriptions, 18 translations, 5 transports, 62 heterodimer association and 5 dissociations (Table F in S2 File). Supporting information for each interaction in the comprehensive map is documented separately in Table G in S2 File.

### Linking lesser-studied/reported molecules with comprehensive map

**Fig 3:**
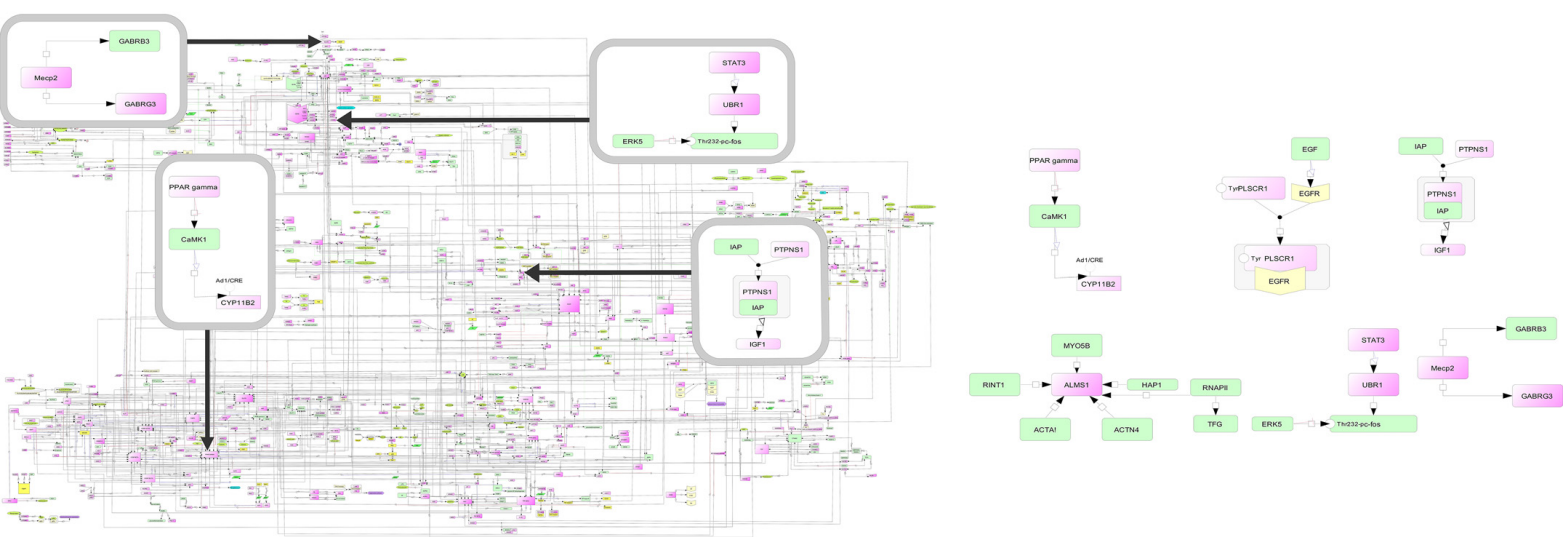
(A) Linking lesser studied (reported) genes (CYP11B2, UBR1, MECP2 and PTPNS1) with molecules of comprehensive map. (B) Examples of lesser studied gene interactions (CYP11B2, PLSCR1, PTPNS1, ALMS1, UBR1 and GABGR3).

There are several clinical conditions as well as syndromes, where obesity is one of the reported phenotype apart from other clinical features characteristic of that syndrome. These include Prader-Willi syndrome, Ulnar-mammary syndrome and Biemond 2 syndrome (See Table E in S2 File). Prader-Willi syndrome is characterised by hyperphagia, characteristic facial features, hypogonadism and short stature. This syndrome is caused by loss of genes imprinted on 15q11-q13 region such as gamma-aminobutyric acid (GABA) A receptor, gamma 3 (GABRG3), imprinted in Prader-Willi syndrome (IPW), small nucleolar RNA, C/D box 116 cluster (PWCR1), small nuclear ribonucleoprotein polypeptide N (SNRPN) and MAGE-like 2 (MAGEL2). We retrieved information for these genes from literature databases as well as from relevant pathways such as KEGG [37] and REACTOME [38]. Then, we aimed to find any evidence of relationship between less-studied genes with obesity network molecules. After extensive manual screening, we were able to find one study[39] which links the **GABRG3** with methyl CpG binding protein 2 (MECP2) gene. The MECP2 gene is a part of module 1 of comprehensive network (See Fig 3). Encouraged by this result, we screened over 6000 abstracts representing 84 lesser-studied genes using our text-mining approach. These includes molecules such as, CYP11B2 (cytochrome P450, family 11, subfamily B, polypeptide 2), PLSCR1 (phospholipid scramblase 1), PTPNS1 (signal-regulatory protein alpha gene interactions), ALMS1 (alstrom syndrome 1), UBR1 (ubiquitin protein ligase E3 component n-recognin 1) and GABRG3 (gamma-aminobutyric acid A receptor, gamma 3). These efforts led to identification of several abstracts/studies through which we could link highly connected nodes with lesser-studied genes with the help of intermediary molecules. For example, we could identify that **CYP11B2** (molecule belonging to lesser studied group) expression and secretion is inhibited by peroxisome proliferator activator receptor gamma (PPAR γ), a key molecule in adipocyte lineage [40] and a reported hub of our map. Additional details are given at our website http://tinyurl.com/knnqsmm. We also computed composite score of lesser studied (reported) genes and compared with hubs (well studied genes) (See File I in S1 File).

### Structure of the map

**Fig 4:**
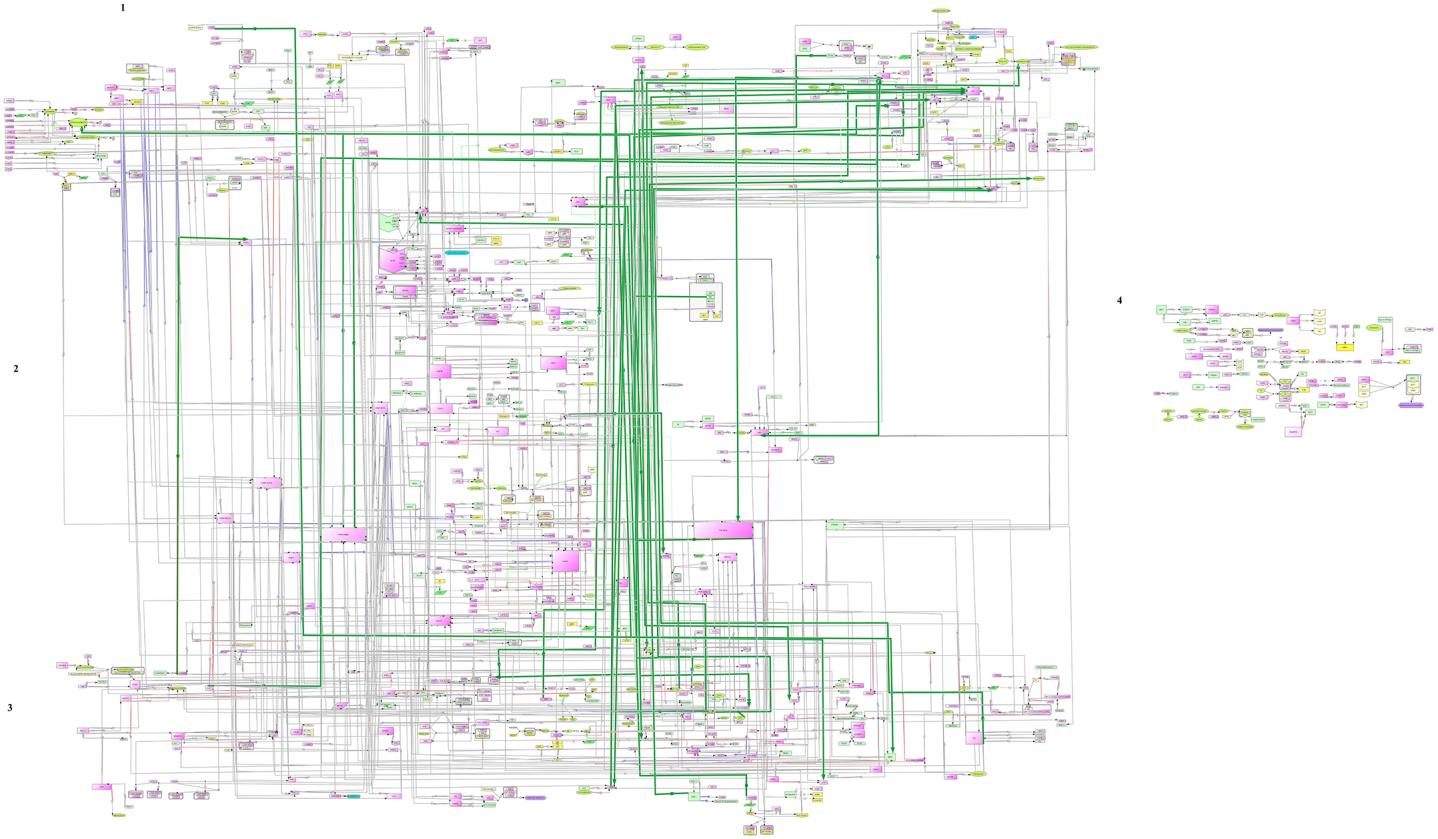
I-shape structure of the network consisting of central component connected with top and bottom regions. The feed-back loops are highlighted in dark green colour.

We used standard techniques to find structure in the constructed map [41]. The map has bow-tie architecture and resembles to alphabetical character "I". To facilitate map exploration, we divided our map into three regions: top, intervening (or central) and bottom. The prominent class of molecules present in the top region include neurotransmitters (catecholamine, dopamine, &serotonin), lipoproteins [lipoprotein lipase (LPL) & high density lipoprotein (HDL)], receptors [peroxisome proliferator-activated receptors delta δ (PPAR δ)], and cytokines [interleukin 6 (IL6)]. This indicates involvement of wide variety of molecules in obesity pathophysiology. The central part includes extensively reported molecules (leptin, ghrelin & insulin) along with less frequently reported molecules such as forkhead box A2 (FOXA2/HNF3B), pancreatic and duodenal homeobox 1(PDX1) and lep-ob-Rb (leptin-leptin receptor) complex (See Table 1). The bottom region majorly consists of transcription factors and signaling molecules, inclusive of glucose transporter 4 (GLUT4), adiponectin (ADIPOQ), lipin1 (LPIN1), fatty acid binding protein 4 (FABP4), necdin, BMP delta-like 1 homolog (DLK1/PREF1), tumour necrosis factor alpha (TNF α) and PR domain containing 16 (PRDM16). In addition, several feedback loops connect top and bottom regions (highlighted in dark green colour in Fig 4).

**Table 1:** Shows three components of the map: top, bottom and central component along with the molecules and their connectivity degree.

I SHAPE STRUCTURE			
CENTRAL COMPONENT		BOTTOM PART	
NODES	CONNECTIVITY DEGREE	NODES	CONNECTIVITY DEGREE
LEP	31	C/EBP ALPHA	24
OB-Rb-LEP	8	C/EBP BETA	18
OB-Rb	2	C/EBP DELTA	14
TYROSINE HYDROXYLASE	5	PREF1	11
GHRELIN-GHSR	8	NECDIN	7
AGRP	8	BMP7	9
POMC	7	BMP2	7
NPY	7	BMP4	7
ALPHA-MSH	6	LPIN1	8
PDX1	8	AGPAT2	5
HNF3B	7	BSCL2	4
FOXO1	7	SREBP1C	6
AKT	6	FABP4	12
SREBP1	6	GLUT4	18
PI3K	5	PPAR GAMMA	41
INSULIN	32	MIR103	6
IRS1	5	PPAR GAMMA 2	6
P38	11	PRDM16	7
**TOP PART**		ASP	5
DOPAMINE	9	TNF ALPHA	18
CATECHOLAMINES	8	PAI1	12
NOR-EPINEPHRINE	6	PGC 1 ALPHA	12
TRYPTOPHAN	3	ERR GAMMA	7
SEROTONIN	6	PPAR ALPHA	9
CCK-CCKAR	3	ADIPOQ	8
EPINEPHRINE	3	NRF2	3
DRAJC3	3	ERR ALPHA	7
ALPHA ADENYL CYCLASE	4	NRIP1	5
SCD	9	TCF	12
MLXIPL	4	CTNNB1	12
LPL	13	TCF4	4
HDL	4	AMPK	8
FXR ALPHA	5		
CHOLESTROL	7		
IL6	11		
CHOLESTROL ESTER	7		
PPAR DELTA	6		

### Representation of the map

Genes and proteins are represented by standard notations, whereas interactions are categorized as positive, negative, neutral and catalysis. A positive regulation is defined for set of molecules, in which the molecule’s activity is stimulated by another molecule. In this context, authors frequently uses specific verbs such as stimulate, activate, induce, enhance, up-regulate and increase. Negative regulation is the inhibition of the neighbouring interacting molecule which is evident by verbs like inhibit, down-regulate, decrease, prevent, suppress and reduce. The edge representations include transcription, translation, association and dissociation using standard graphical notation. Apart from these, there are some reactions where a molecule regulates the reaction between other molecules, i.e. catalysis (See Table 2). The colour scheme and graphical representation is explained at website (http://tinyurl.com/dykn8fd) and File J in S1 File.

**Table 2:** Shows types of interaction, example of verbs, representative sentences and references.

Types of interaction with verbs	Sentences	References
**Positive**		
Activate	Leptin in central nervous system exhibit their action by binding to ob-Rb, a long isoform of leptin receptor. The binding leads to the ***activation*** of four tyrosine residues by phosphorylation and a Box 1 region in ob-Rb.	Frühbeck,2006
Positive	The STAT3 exhibits a ***positive*** regulation on POMC [pro-opio melanocortin], which are the major molecules in energy regulation	Frühbeck, 2006
Induce	C/EBP β and C/EBP δ ***induces*** kruppel-like transcription factor [KLF] 5.	A. Schäffler et al, 2006
Stimulate	E2F1 ***stimulate*** PPAR γ during early stages and E2F4 inhibit it in later stages.	Fajas, L. et al, 2002
Up-regulate	In adipocytes, SORBS1 is ***upregulated*** by thiazolidinedione [TZD] through PPAR γ and also interact with insulin receptor.	Wen-Hsing Lin et al, 2001
Increase	PRDM16, which leads to ***increase*** in expression of UCP1, PGC α and PGC β during mature adipocyte development	Yu-Hua Tseng et al, 2008
Enhance	Urocortin ***enhances*** Leptin-Induced Stat1 Activation, Mediated by Either CRHR1 or CRHR2.	Weihong Pan et al,2007
Require	We report that exercise and recombinant IL-6 requires IL-10 expression to suppress hyperphagia-related obesity.	Eduardo R. Ropelle et al, 2010
Bind	Follistatin binds to myostatin but also binds to and inhibits other members of the TGF-beta superfamily, notably activins.	Nakatani M et al, 2008
Trigger	AMPK activated through leptin and external stimuli, ***triggers*** myocytes enhancing factor 2 [MEF2] [A & D] which leads to the expression of GLUT4.	Farid F. Chehab, 2008
**Negative**		
Inhibit	PPAR γ ***inhibit*** the expression of aromatase by CYP19A1 gene.	Reposo Ramirez-Lorca et al, 2007
Negative	LXR [liver X receptor], a transcription factor take part in adipogenesis by ***negatively*** regulating PGC-1, glucose-6 phosphatase, phospho*enol* pyruvate carboxykinase [PEPCK/PCK2].	Bryan A. Laffitte et al, 2003
Prevent	PON3 in plasma individually ***prevents*** artherosclerosis by inhibiting phospholipid stimulation of monocyte chemotactic protein [MCP]	Diana M. Shih et al, 2007
Decrease	PPAR δ ***decreases*** LDL and triglycerides.	Markku Va¨nttinen et al, 2005
Reduce	In the presence of ID3, the binding of E47 and SREBP1 is ***reduced***, thereby transcriptional activity of ADIPOQ is controlled.	Curt D. Sigmund et al, 2008
Suppress	In conclusion, disruption of neuromedin B receptor did not interfere with the sensitivity of thyroid hormone-mediated ***suppression*** of TSH release, but impaired the ability of thyrotroph to increase serum TSH in hypothyroidism, which highlights the importance of NB in modulating the set point of the hypothalamus–pituitary–thyroid axis at hypothyroidism.	Karen J Oliveira et al,2008
Down-regulate	We demonstrate that activation of LXR in the liver leads to the induction of glucokinase expression and to the ***down-regulation*** of peroxisome proliferator-activated receptor γ coactivator-1α [PGC-1] and genes involved in gluconeogenesis.	Bryan A. Laffitte et al, 2003
**Neutral**		
Regulate	The cyclic AMP responsive element–binding protein-1 [Creb1]-***regulated*** transcription coactivator-1 [Crtc1] is required for energy regulation	Judith Y Altarejos et al, 2008.
Correlate	In girls, visfatin ***correlated*** with leptin, r = 0.40, P = 0.009, and thiols, r = -0.36, P = 0.009, which explained 24% in visfatin variability.	Krzystek-korpacka M et al, 2011
Elicit	At basal glucose, GIP does not ***elicit*** insulin release.	Pfeiffer AF et al, 2010
Influence	An exciting new report describes that leptin can ***influence*** insulin release by osteoclastin, a hormone produced by osteoblasts.	Rashmi C and Rodger AL, 2011
Associate	Leptin was ***associated*** with insulin, insulin resistance, and body composition parameters, body mass index, basal metabolic rate, body weight, %fat, and fat mass, in participants, with or without T2DM in both genders.	Gulturk S et al, 2008
Affect	In conclusion, HSL ***affects*** insulin secretary capacity especially in the setting of obesity.	Sekiya M et al, 2009
Contribute	FOXO1 may ***contribute*** to enhanced ADIPOR1, but not ADIPOR2 transcription in IR.	Felder TK et al, 2010
**Catalysis**		
Catalysis	Tyrosine hydroxylase [TH] ***catalysis*** the conversion of L-tyrosine to DOPA which in-turn is regulated by SPOCK1, fgf14 [fibroblast growth factor-14], Cxcl14, BMP6 [bone morphogenetic protein 6]	C. Vadasza, 2007

### Module generation—Reverse Engineering of the pathway

**Fig 5:**
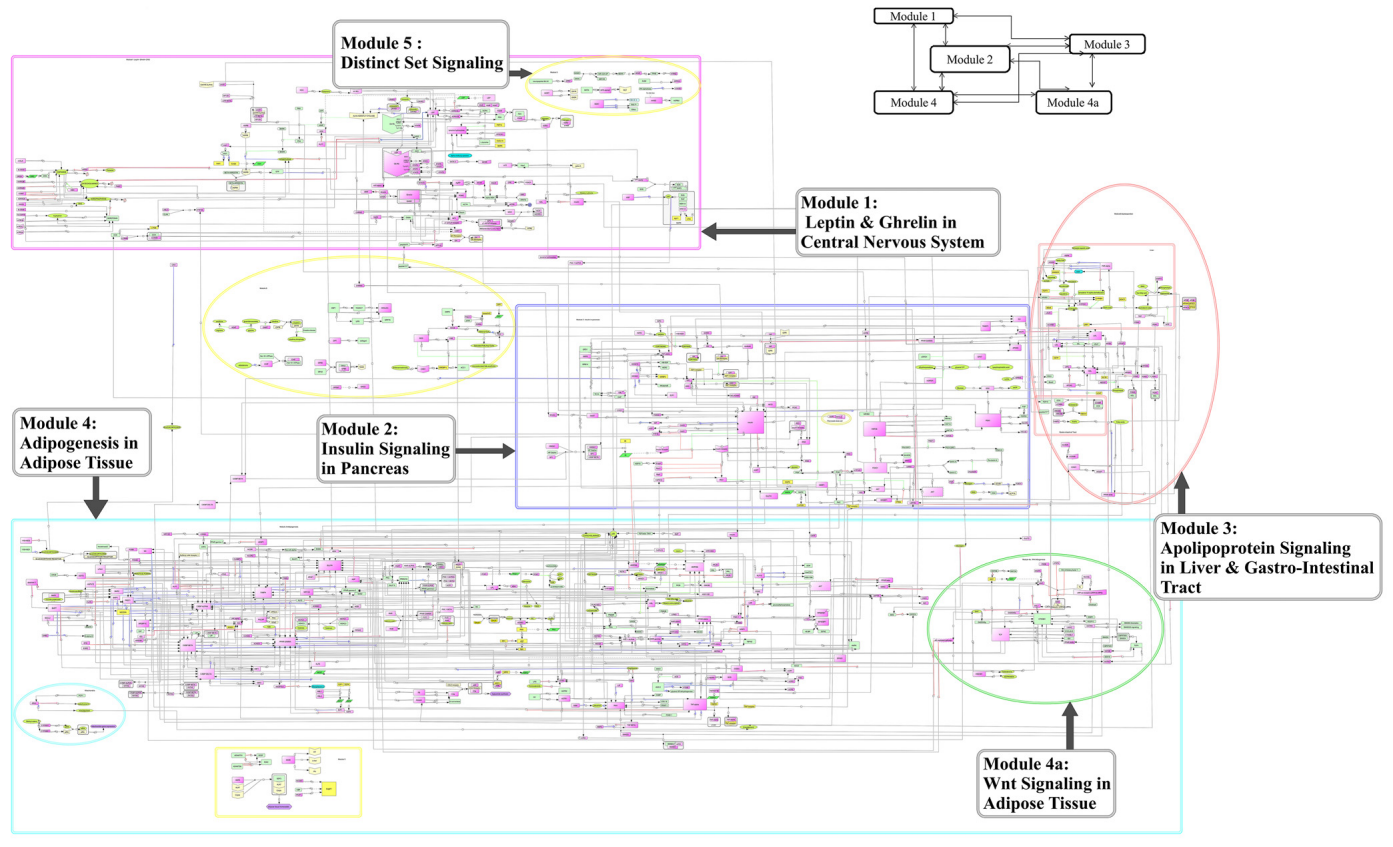
Modules of the comprehensive map.

To understand a large network, a logical step is to divide the network into biologically meaningful smaller functional components [42]. This process is often termed as reverse engineering and several approaches have been described to identify modules. These range from spectral methods [43–44], methods that identify maximum flows or minimum cuts [45–46], heat kernels [47], betweeness centrality [48], seed node searches [49] e.g. MCODE in cytoscape [50], brute force methods [51] and weighted kernel k-means [52]. *Community structures* or *modules* are defined when a larger density of links exists within a specific part of the network than outside it [53]. We used different methods to identify community structures (modules) in obesity network. In addition, we clustered genes based upon tissue specific expression data. Since each method produces different results with some degree of overlap, we decided to integrate information to identify functionally meaningful modules (See File A in S1 File). Hence, the constructed network was divided into 5 modules based upon physiological processes and likely anatomical component (Table 3). In the following section, we attempt to relate modules with disease conditions (Fig 5).

**Table 3:** Describes information of five modules obtained from the network. The columns show pre-dominant hub, likely anatomical component and physiological process with the connectivity degree of major molecules.

Modules	Pre-dominant Molecule	Likely Anatomical Component	Physiological Process	Major Molecules with their degree of connectivity
Module 1	Leptin	Central Nervous System	Satiety, Appetite, Energy expenditure	Leptin [LEP]– 14, Dopamine– 9, Catecholamine– 8,Ghrelin—Growth Hormone Segretagogue Receptor[GH-GHSR]-5, Agouti-related Protein [AGRP]– 8, Pro-opiomelanocortin [POMC]– 6, NeuroPeptide Y [NPY]– 7,Tyrosine Hydroxylase—4, Adregenic Receptor Alpha 1B [ADRA1B]-3
Module 2	Insulin	Pancreas	Glucose metabolism	Insulin [INS]– 21,Forkhead box A2 [HNF3B]– 6,Pancreatic and Duodenal Homeobox 1 [PDX1]– 7,Forkhead box O1[FOXO1]– 6,Interleukin 6 [IL6]– 5 Leptin [LEP]– 5,Insulin receptor substrate 1 [IRS1]– 6, Insulin receptor substrate 2[IRS2]– 5,Insulin receptor– 5
Module 3	High Density lipoprotein	Liver & Gastro-Intestinal Tract	Fatty acid regulation and metabolism, Base-line glucose control.	Lipoprotein Lipase[LPL]– 6,High Density Lipoprotein– 12,Low Density Lipoprotein– 6,FXR alpha– 5,Cholesterol– 6
Module 4	PPAR gamma	Adipose Tissue	Store fat derived from the diet and liver metabolism or degrades stored fat to supply fatty acids and glycerol to the circulation.	Peroxisome proliferator-activated receptor PPAR alpha– 9,PPAR gamma– 41, PPAR beta– 3,PPAR gamma 2–6, PGC1alpha -12 CCAAT/enhancer binding protein C/EBP alpha– 24,C/EBP beta—18 C/EBP delta– 14,Glucose Transporter 4–9,Tumor Necrosis Factor Alpha-18, Adiponectin [ADIPOQ]– 8,PR domain containing 16–7,PREF1–11,Necdin– 7 Lipin1- 8,Sterol regulatory element binding transcription factor 1 SREBP1c- 6
Module 4a	CTNNB1	Adipose Tissue	Inhibitor of Adipogenesis	Catenin [cadherin-associated protein], beta 1 CTNNB1–12 HNF1 homeobox A [TCF]– 9
Module 5	Distinct SetsSignalling	--------	--------	---------

**Module 1**: This module consists of highly connected nodes involved in neuro-hormonal signaling affecting energy homeostasis, hunger and mood. They include leptin, ghrelin and dopamine. Leptin is one of the highly studied molecules in obesity after insulin (present in7.4% of total abstracts). Leptin acts as a satiety factor and its discovery has paved the way for the study of adipocyte derived factor in energy balance homeostasis. Further, the secretion of the leptin is directly proportional to amount of fat cells [54]. Recently, leptin replacement therapy has been proposed to treat obese individuals[55]. Frequent association of obesity with clinical depression can be explained by the impaired leptin activity in brain [56].

Ghrelin act as an endogenous ligand for growth hormone secretagogue receptor (GHSR). It has been reported to be involved in energy regulation and appetite signaling through activation of peptides, including AgRP, NPY and POMC [57]. Rise and fall in plasma ghrelin levels before and after food intake supports the hypothesis that ghrelin plays a physiological role in meal initiation in humans [58]. Ghrelin levels are altered in individuals suffering from Prader-Willi and Cushing's syndrome [59]. A meta-analysis linked gastrointestinal hormones, ghrelin and obestatin levels with obesity [60].

The importance of dopamine signaling in obesity has been demonstrated by the alteration of dopamine receptor levels with changes in body mass index (BMI) [61]. Apart from these, several other molecules have been reported in context of obesity; therefore, we have described their roles at our website: http://tinyurl.com/kazahj6.

**Module 2:** Obesity is a major risk factor for non-insulin dependent diabetes mellitus (NIDDM) [62]. Insulin is a central molecule in pathophysiology of type 2 diabetes and also appears in large number of abstracts related to obesity (23,165 abstracts; 24% of total dataset) in humans. Module 2 primarily encapsulate insulin and its interactions with other molecules, for instance, apolipoprotein A-V (APOA5), forkhead box C2 (FOXC2), macrophage migration inhibitory factor (MIF), uncoupling protein (UCP) and v-akt murine thymoma viral oncogene homolog 2 (AKT2). This module builds a link between tightly coupled clinical conditions- obesity and type 2 diabetes.

**Module 3**: Lipid storage and metabolism is affected frequently in obese patients leading to dyslipidemia, exposing them to cardiovascular risks [63] and atherosclerosis [64]. The third module maps interactions, catalysis and processing of molecules involved in lipid metabolism, including acetyl CoA, aspartate, mevalonate, cholesterol, cholic acid, and diacylglycerol.

**Module 4**: It is the largest module in the network and majorly consists of transcription factors involved in adipose tissue differentiation and other biological activities in humans. The interactions are dominated by molecules like peroxisome proliferator-activated receptors-PPAR (α, β, γ) and CCAAT/enhancer-binding proteins-C/EBP (α, β, γ). The molecules such as PPAR γ (with 41 edges) provide indirect connections with lesser studied genes/molecules reported in context of obesity. This module is divided into another sub-module labelled as “4A” to incorporate set of molecules distinct from transcription factors.

**Module 4A**: Though the Wnt pathway has been shown to play a major role in embryogenesis and some of the cancers, it has also emerged as an important regulator of adipocyte differentiation [65]. In addition, recent evidence of obesity treatment using traditional herbal medicine, SH21B, has indicated about anti-adipogenic mechanism mediated by Wnt-β catenin signaling [66].

**Module 5**: The last module contains information about disjoint set of genes/proteins involved in obesity which are difficult to categorize due to inadequate information.

### Quantitative Analysis

To understand the properties of constructed network, we computed several topological parameters as described below (See File B in S1 File for detailed information).

1. **Degree distribution parameter**: We found that the several number of connections follow power laws that indicates scale-free pattern of connectivity (γ_in:_ in-degree parameter as 2.19 and γ_out_: out-degree parameter as 2.11). The scale free behaviour is also observed in constituent modules suggesting preferential attachments and hubs in the network (See Table 4).
2. **Clustering Coefficient:** Our network and its constituent modules show clustering coefficient values and average clustering coefficient [67] close to 0 suggesting tree-like structure [68].
3. **Average shortest path length** value was found to be 15.85 for comprehensive network supporting scale free nature of the graph [69].

**Table 4:** Topological analysis of the comprehensive map using Network analyzer and Gephi.

S.No.	Parameters	Network Analyzer	Gephi
1	Nodes	1799	1799
2	Degree Distribution		
	In-degree		
	γ	2.193	-
	R^2^	0.866	-
	Out-degree		
	γ	2.116	-
	R^2^	0.914	-
3	Clustering Co-efficient	0	-
4	Connected Components		
	Weakly Connected	35	35
	Strongly Connected	-	1398
5	Diameter	46	46
6	Average Shortest Path-length	15.86	15.86
7	Average number of neighbours	2.37	
8	Network Density	0.0	0.001
9	Average Degree	-	1.192
10	Average Weighed Degree	-	1.6
11	Modularity	-	0.875
12	No. of Clusters	-	62

### Randomization of Constructed Network

We constructed null models (control) and compared the properties of comprehensive network with null models [68, 70]. The protocol is described as following: In a true network gene A (leptin) binds with gene B (leptin receptor) to perform a function X (i.e. leptin act as satiety factor and exhibits its action by binding to leptin receptor) in cell.

Null model 1- In this model, we randomised the edges but kept the node labels and their degrees intact. For example, the connection (edge) between gene A (leptin) and gene B (leptin receptor) is deleted. A new connection is established between gene A and gene C (any other gene of the network except leptin receptor) so as to disrupt the function X.

Null Model 2- We shuffle the positions of nodes by keeping the global degree distribution of the comprehensive map intact.

Null Model 3- This is generated by shuffling both the position of nodes as well as their edges (See File C in S1 File).

Null Model 4- We construct the network with same number of edges and nodes using methods proposed by Erdos-Renyi [71], Watts-Strogatz [72] and Barabasi-Albert [73]. To see the effect of properties on size of the network, we construct networks with node numbers from 100 to 1000. Firstly, we use method proposed by Erdos-Renyi to construct a random graph of N nodes connected with n edges, which are chosen randomly from N (N-1)/2 possible edges and are not scale-free [71]. Secondly, a control network was generated through Watts-Strogatz model (1998) [72], where in random graph is produced with small-world properties, including short average path length and high clustering. Thirdly, in Barabasi-Albert model [73], the generation of random graph is based on the connected seed network of s nodes. Remaining nodes (n-s) are added one at a time, and connected to existing nodes (m) randomly. The resulting network is found to follow power-law degree distribution.

In addition, we generated randomized networks using random network module of cytoscape. The obesity network (true network) exhibit different properties when compared to 18 control randomized networks obtained by shuffling the obesity network associations while keeping the degree distribution of nodes fixed (Fig G and Fig H in S1 File). We find that clustering coefficient increases from 0 (in true network) to 0.00201 (randomized network with 30000 shuffling. See Fig G in S1 File). This pattern is reversed in case of mean shortest path, which reduced from 18 to 11 units (See Fig H in S1 File). We have also enclosed additional information for results generated during shuffling procedure in the Table H in S2 File and website in S3 File.

### Robustness of Network

To see the robustness of network and its dependence on failure of a particular node, we randomly deleted nodes and computed properties for the remaining network. There are several indexes of network centrality such as degree, eccentricity, closeness, betweenness, stress, centroid and radiality which allow quantifying the topological relevance of single nodes in a network. Recently introduced parameters such as node interference and robustness were also included in the analysis. These parameters measure the relative importance of given node in context of network [74]. It was also shown in the past that the hubs (nodes with high degrees) play important roles in maintaining structural integrity of networks against failures and attacks, [75] in spreading phenomenon [76] and in synchronisation [77]. Since, obesity network shows scale free structure with presence of hubs, we started our deletion experiments by sequential deletion of hub nodes to see the effect on network robustness. This was achieved by removing a node and calculating the interference on the centrality of the remaining nodes using centiscape plugin of cytoscape. We find that removal of hubs alone or in combination impact the network tremendously. We find that various critical properties of network changes to significant extent. For example, betweenness of nodes of original network (Mean = 10738.4; Var. = 3.3E8) were significantly different when compared with networks obtained after deleting all hubs (Mean = 13675.4; Var. = 1.1E9) computed through paired t test (P<0.05). When we randomly deleted any node (not hub), the changes were not significant in the parameters (See Table I in S2 File and Folder: Deletion experiment on website (A) in S3 File).

### Gene Ontology Analysis (GOA)

To understand the biological processes present in large dataset of obesity genes, we used BiNGO [78] and Network Ontology Analysis (NOA) [79]. We observed that adipocyte specific functions, including response to nutrient level were represented by 48 molecules (10% of total dataset, p value: 2.49e-25), regulation of lipid metabolic process was seen with respect to 47 molecules (9.9% of total dataset, p value: 2.44e-37), carbohydrate metabolic process (38 molecules; 8%, p value: 9.5e-07), lipid localization (36 molecules; 7.6%, p value: 3.75e-22), lipid biosynthetic process (35 molecules; 7.3%, p value: 5.42e-10), feeding behaviour (31 molecules; 6.5%, p value:8.0e-30), and response to nutrient by 31 molecules (6.5%, p value: 2.49e-25). In biological process, the sub-category- cellular process comprises 80.9% of the genes of our dataset, which include cell communication (83 molecules; 22.6% of total dataset, p value: 1.06e-22), regulation of gene expression (152 molecules; 41.5%, p value: 4.43e-12) and regulation of programmed cell death (72 molecules; 19.6%, p value: 6.89e-15). Similarly in molecular function, 92.9% genes are involved in binding activity and in cellular process/location- 69.5% of the genes are found to be present in intracellular section of cell which primarily includes nucleus (165 molecules; 59.1% of total dataset, p value: 1.03e-02) and endoplasmic reticulum (50 molecules; 17.9%, p value: 4.33e-05) (See Table J in S2 File).

### Mapping of Microarray Data

The microarray data was obtained from Gene Expression Atlas [80] using search term “obesity and homo sapiens” from the URL (http://www-test.ebi.ac.uk/gxa/). The gene list was obtained for three possible conditions: up-regulation, down regulation and non-differentially expressed. We selected 3,485genes reported to be up-regulated in obesity and labelled them as set ‘U’. Subsequently, we found 2,135 genes (labelled as D) as down-regulated group and a very large number of genes (2,91,407) as non-differentially expressed (NDE). After removal of redundancy, we obtained 1,340 molecules as up-regulated (U), 918 molecules as down-regulated (D), and 38,434 molecules as non-differentially expressed (NDE) molecules as a filtered sets.

Thereafter, we compared filtered dataset obtained from microarray database with our list. Based upon comparisons, we found that 27 genes (obtained from deep curation approach (DC)) are up-regulated in obesity whereas 24 genes show down-regulation and large numbers of genes did not show any change in expression or information is not available in the database. Using gene ontology analysis, it was revealed that most of the up-regulated genes are involved in protein binding and down-regulated group are involved in steroid binding activity (See File D in S1 File).

Since, we could not map large number of genes; we attempted to find expression data of obesity genes in GEO (http://www.ncbi.nlm.nih.gov/geoprofiles) microarray database. We found that 34.5% of genes (obtained from text mining (TM) approach) are up regulated whereas 27.58% are down regulated.

### Applications of Obesity Network-Implications in therapeutics

We used orlistat (tetrahydrolipstatine, an FDA approved drug for treatment of obesity) to dock against the molecules listed in our network using our in-house docking pipeline “Docoviz”. We observe that orlistat not only binds to fatty acid synthase (FASN) (ΔE = -13.7 Kcal/mol; experimentally known target) but also binds to several other molecules in the obesity network. To check whether orlistat produces its clinical effect (of weight reduction) possibly due to preferential binding to several molecules listed in the obesity network (N) than any other part of proteome, we created a dataset of 24,000 known human protein structures (P) and docked orlistat against them. In addition, we created datasets of randomly selected protein structures from P labelled as P1, P2…Pn as controls. We also used Alzheimer disease network molecules [81] as an additional control (D). We observed that the distribution of binding energies obtained from controls (P1, P2, P3…Pn) and Alzheimer disease network(D) is significantly different from test dataset(N) (P value <0.05, Welch T test).

In another experiment, we docked drugs (which do not have effect on obesity) against the obesity network proteins. For instance, we used Acetylsalicylic acid (selected randomly; anti-inflammatory medicine) to dock against the obesity network proteins. Apart from that, we used drugs, showing comparable tanimoto co-efficient to orlistat, such as 3-Carboxy-N,N,N-Trimethyl-2-(Octanoyloxy) Propan-1-Aminium (Tc Value: 0.68) and 6-DeoxyerythronolideB (Tc Value: 0.6) to ascertain binding energy profiles in the obesity network. We detected that the binding energy profiles of the above mentioned drugs against the obesity network proteins are different from that of orlistat (P value <0.05, Student’s T test).

Orlistat is known to produce several side-effects namely acne, respiratory tract infection, urinary tract infection and nausea, possibly due to binding to off targets perturbing unrelated pathway. Using text mining systems and manual screening, we obtained list of molecules implicated in the side effects/diseases related to orlistat. On comparison, we found that several molecules are common in obesity network and acne (14 molecules; 2.7% of total dataset), providing a possible clue for causation of acne in patients taking orlistat during obesity treatment. Likewise, sibutramine (antidepressant and anorexigenic drug) was withdrawn due to adverse effects such as agitation, fever, vomiting, diarrhoea, loss of coordination, and dilated pupils. Using our map, we could link the side effects of sibutramine with disease networks. To illustrate, symptoms such as nausea, vomiting and depression are likely to be produced due to binding of sibutramine to targets such as SLC6A3 and SLC6A4 and subsequent perturbation of pathway involving HTR2C (anxiety), HTR2A (anxiety), DRD2 (nausea and vomiting), COMT (nausea and vomiting), and MAOA (depression) (File E in S1 File).

## Discussion

This work shows a new approach of combining data from heterogeneous databases including literature, structure and microarrays to construct disease networks and attempt to explain therapeutics of a drug molecule in context of networks. Our methods are generic, web enabled and open in nature to build rich networks. Each entity i.e. node or edge has been hyper-linked to its source (research papers) so as to maintain transparency in the system for users to evaluate and improve the system in a collaborative fashion.

Network targeting involves activity of a compound across multiple pathways which might be necessary to effectively stop neoplasm and pathogens, but can also produce side effects by targeting undesirable proteins [82]. Very few large scale docking studies have been conducted in the past (Gao *et al*. used ~1,100 targets [83]; Hui-fang *et al*., used 1,714 targets [84]; [85]). Here, we performed docking of orlistat with obesity network proteins as well as with whole human proteome (>24000 proteins) as a test example. Based upon our predictions, we propose that a given drug (orlistat) not only bind to its known target (FASN; ΔE = −13.6 Kcal/mol) but also to several other targets in the network with varying degree of binding energies. This propensity of binding of drug within the target network (obesity) is different from binding with any other disease network or network randomly drawn from human proteome. Further, we also observe that the therapeutically unrelated drugs for a given clinical condition (“Acetylsalicylic acid in obesity”) show different binding patterns to network proteins. These results contribute to emerging concepts of network pharmacology [82] and chemigenomics [86] to develop safer, cheaper and effective medicines. The possible limitation of this approach is non-specific or random binding of ligand to many of the protein targets.

Real world networks including biological networks are characterised by presence of few highly connected nodes known as hubs and they tend to show non-Poisson degree distribution. Evidence shows that hub proteins are encoded by essential genes [87] that seem to be older, evolve slowly and their deletion affect a large number of nodes as compared to non-hub nodes [88–90]. Therefore, different studies have attempted to associate hub proteins to disease genes. Some studies support this hypothesis, whereas few studies contradict this hypothesis [90–92]. Our network shows hub based architecture with select set of nodes occupying most of the connections- leptin, insulin and PPAR gamma. Most of these genes likely to be essential in nature, whereas some of the recently reported candidate genes are present in periphery in our map, e.g. fat mass and obesity associated (FTO) gene. It may be inferred that the obesity pathophysiology is primarily influenced by interactions of essential genes, therefore obesity could be considered as a system level adaptation toward chronic nutritional over intake and other causative factors.

We compared our network with previously published dataset, including Kitano *et al*, 2004[41], Logsdon et al, 2012 [93] and found several of our network molecules present in these datasets (See File F in S1 File). Various population wide studies have indicated that hypertension is a predominant clinical condition affecting over 40% of obese people (BMI > 30) [94], whereas type II diabetes mellitus affects 40–60% of obese people [95]. Using text mining approaches, we found that there is a significant overlap between molecules implicated in obesity and its associated disorders such as diabetes or hypertension. This overlap is less when molecules implicated in obesity are compared to molecules implicated in unrelated disease group e.g. asthma, urticaria and ataxia.

Considering wide variety of factors affecting the obesity pathophysiology, we believe that obesity comprehensive map will act as a platform to integrate information derived from gene expression experiments, protein-protein interaction data, drug information, clinical data, metagenomic and pharmacogenomic information. It will be interesting to understand how this network evolves temporally in a lifespan of a given individual(s) from lean state to obese state. What modules or links get formed or abolished during the process? It can also act as a system where new drugs may be tested against disease networks to predict their therapeutics or side-effects.

## Material and Methods

### (A) Retrieval of Literature Data

We screened each research article manually and highlighted text for the name of molecules as well as their interactions. We also used information provided in human obesity gene map database 2005 update [30] and GenMapp (http://www.genmapp.org/default.html). The abstracts having the term “obesity” and “human” were downloaded from PubMed using RefNavigator (version 2.0). We obtained 96,219 abstracts on obesity in human till December 2012 (See Folder 2 available at website (A) in S3 File). We used perl scripts to parse additional information which includes authors’ names, affiliations, journal name & year of publication. Each abstract was processed and unique id was assigned using perl scripts.

### (B) Determining True Positives and False Positives

Researchers have used several approaches to link genes with complex traits such as obesity. Primarily, linkage analysis and association studies have been used to find the variants that affect obesity. In addition, animal models also provide list of candidates genes through linkage studies, expression profiling, and transgenic strains. The techniques such as expression analysis and protein interaction studies also identify candidate genes for obesity. Given the wide variety of available experimental techniques, we grouped these studies (evidences) into various categories and provided a numerical code to each of them (See Table B in S2 File). Next, we label each gene with a numeric code for better data management.

A gene is defined as true positive example, when we have enough evidence to link a gene with a disease. For example, Leptin (Lep) deficiency is linked with intractable form of obesity (Uniprot Id—P41159; OMIM ID- 614962). As a rule of thumb, we labelled genes with high confidence when many independent research studies published in high impact journals with sufficient citations support that link. Since, each gene has different types of experimental evidences ranging from mutation studies, animal studies, genome wide association linkage studies and clinical studies. We grouped these evidences into various categories and provided a numerical code (See Table B in S2 File). The false positives are those gene examples which matched common English words used in sentences, abbreviations of organizations, and author names. They also include examples which occurred in abstracts but rejected during manual screening due to lack of clear evidence.

### (C) Hybrid approach

Deep-curation approach (DC) is defined as screening of literature data by experts whereas text-mining systems (TM) sift through publication data for the occurrence of the genes and their interactions using computational software and predictive algorithms at large scale. Though, text mining systems are fast, but they suffer from several problems limiting their use. For example, consider a representative statement from a research article [54], “the binding of the SH2 domain of SH2B1 to phospho-Tyr, 813, in JAK2 enhanced leptin induction of JAK2 activity”. Here, different text mining tools will report—“Jak2 enhanced leptin”. This is considered to be a positive interaction but the real meaning is leptin increases JAK2 activity upon binding of SH2 domain to JAK2. Due to these constraints, text mining systems are not considered robust enough to resolve numerous problems warranting the need for deep-curation approach. Our TM approach is formulated as following-:

(i) Let *W* be the set of all the genes and their synonyms in human that may occur at least once in the set of abstracts labelled as *A*. The *W* is represented as a matrix where each row represents a gene (*w*_*i*_) and its synonyms. The synonyms and approved symbols for each gene are shown in tab separated format in a text file where notation “*w*_*ij*_” is designated for them.
(ii) A separate matrix (*M*) is constituted for storing frequencies of genes, listed in *W*. It contains genes (*w*_*i*_) in the first column and their respective counts (*c*_*k*_) in second column. For example, *w*_*2*_ represents the gene LEP, having a gene count (*c*_*2*_) of 7,159 in the PubMed abstracts (1960–2012 December).
(iii) We also define *N* asthe gene co-occurrence matrix. Each entity of this matrix is described as *N*_*xyz*_ to store information extracted from research articles. This is composed of three units: *N*_*x*_, *N*_*y*_ and *N*_*z*_. *N*_*x*_ capture first instance of gene encountered in the sentence whereas *N*_*z*_ keeps the next instance of gene and *N*_*y*_ stores intermediate set of words. To illustrate, consider a statement, “**Insulin** is known to increase expression of the *ob* gene product **leptin** in adipose tissue”. Here, insulin and leptin are labeled as gene pair having 10 intermediate words between them. Therefore, “insulin” will be *N*_*x*_; “leptin” will be *N*_*z*_ and “is known to increase expression of the *ob* gene product” is *N*_*y*._
  $$MatrixN=\left|\begin{array}{ccc} Gene1 & InterveningWords & Gene3\\ N1 & N2 & N3\\ N3 & N5 & N1\\ N4 & N6 & N1 \end{array}\right|$$

We extract gene pairs from the abstracts and full length articles and compute their frequencies. We also build frequency distribution of intermediate words (*N*_*y*_) useful for building dictionary for subsequent natural language processing. This dataset is also useful for training of machine learning systems such as hidden markov models and support vector machines (manuscript in preparation) as well as manual curation.

(iv) Parser is a set of dictionaries that are built for various types of interactions, tenses and negations. We curated data of 300 research articles to identify the most frequently used words to represent interactions namely, positive, negative and neutral. We use these dictionaries to label interactions by building a matrix *O*. In matrix *O*, *O*_*xyz*_ represent the data structure where the gene *O*_*x*_ (insulin) is followed by gene *O*_*z*_ (leptin) with their type of interaction, *O*_*y*_ (positive). This is processed for graphical-view using GraphViz (Version 2.28). The detailed example (tutorial) of TM approach is provided in a S7 in S1 File.

### Text-Mining Approach Algorithm

 Let abstracts = *A*;

 Let genes = *W*; // 35,000 Genes in human & its synonym

 Let gene count matrix = *M*;

 Let co-occurrence matrix = *N*;

 Let NLP matrix = *O*;

**for** i = 1 to *n* do // ‘i’ is a row representing a gene in *W*

**for** j = 1 to *n* do // ‘j’ is a column representing a gene name, symbol in *W*

    Let c_k_ = 0; // Initializing the count of a gene ‘i’ in abstracts *A* as 0

**if** i,j ϵ *A* then

      write i to *M*; // write the gene ‘i’ in gene count matrix *M*

      c_k_ = c_k_ +1;

    append c_k_ to *M*; // The gene ‘i’ is appended with its count c_k_ in *M*

     next; // Search for the next gene

  read gene x ϵ *M*; //Reading the gene x from gene count matrix *M*

   read gene z ϵ *W*; //Reading the gene z from the dictionary *W*

    gene-pair N_x,z_;

for x = 1 to n do // x represents the first gene of a gene-pair in *M*

     for z = 1 to n do // z represents the second gene of a gene-pair in *W*

      Let N_x,z_ = 0; // Initializing the count of gene-pair x, z as 0

      Let y = 0; // y is words between gene-pair initialized as 0

       for gene x ϵ *A* do

        for gene z ϵ *A* do

         if x then z then

          read y in *A*

           if length y > 3;

            write x,y,z to *N*; // N_x,y,z_ is a co-occurrence matrix

            N_x,z_ = N_x,z_ + 1; // Total occurrence of a gene pair

            append N_x,z_ to *N*

       next; // Search for the next gene-pair

Let NLP Parser = *P*; //Set of Dictionaries *P*

    Let Interaction verb dictionary = *P*_*a*_; //Sub-dictionary in *P*

    Let tenses = *P*_*b*_; //Sub-dictionary in *P*

    Let negations = *P*_*c*_; //Sub-dictionary in *P*

   for *P*_*a*_ ϵ *N* // Search for interaction verb in *N*

    for *P*_*b*_ ϵ *N* // Search for tenses in *N*

  for *P*_*c*_ ϵ *N* // Search for negations in *N*

     write *O*; // *O* is a NLP matrix

### (D) Comprehensive Map Construction

The comprehensive map of molecules in obesity was constructed using Cell Designer software [35]. Cell Designer support systems biology graphical notation (SBGN) and provides various functions to the users to represent molecular entities, including gene, protein, and RNA as well as edge notations-transcription, translation, inhibition and stimulation. The activity as well as modulation in the molecule can also be represented. The constructed map can be exported as systems biology mark-up language (SBML) format, preferred for computational models of biological processes.

### (E) Module Generation

Reverse engineering of the comprehensive map was conducted using tools and methods mentioned in A File in S1 File.

### (E) Random Model Generation

Random models of the comprehensive map were generated by two approaches: Firstly, by Degree Preserving Random Shuffle using Network Analyzer Tool [96] and secondly, by applying Scale-free random graph (a cytoscape plug-in Random Networks). We also used perl scripts developed in-house for randomisation process.

### (F) Comprehensive Map Analysis

**Fig 6:**
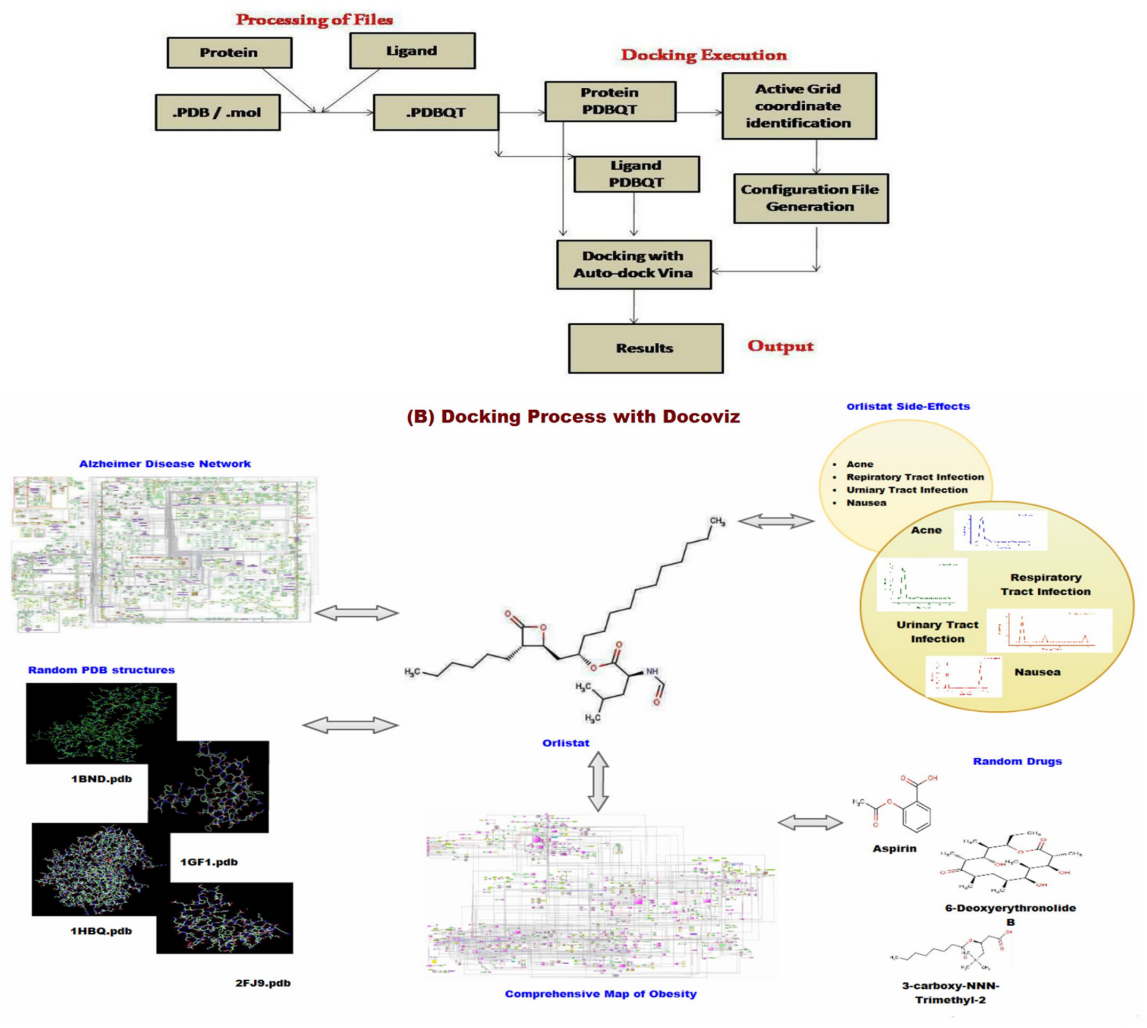
Shows the (A) schematic diagram of Docoviz pipeline and its (B) applications.

1. The topological analysis was performed through graph based algorithms such as NetworkAnalyzer [97] and Gephi (https://gephi.org/).
2. The gene ontology (GO) analysis was carried out for three categories: molecular function, biological process and cellular component using BiNGO [78] and Network Ontology Analysis (NOA) [79].
3. The identification of protein targets of drugs, particularly orlistat, was accomplished with Docoviz pipeline (Fig 6). Docoviz is an automated system used for docking of drugs against protein structures at large scale using Auto-dock Vina [98]. This system is based upon perl and other languages such as ruby (manuscript in preparation). We obtained structural information of the genes implicated in obesity from protein data bank (PDB). Orlistat as well as other drugs were obtained from Drugbank [99] and their side effects were retrieved from SIDER database. The pdb format of protein structure was converted to pdbqt format before commencing the docking procedure. We identified active site coordinates through geometric search method. A grid of about 20Å around the active site coordinates was generated to search all possible transition point (See K File in S1 File).

## Supporting Information

S1 File (DOC)

S2 File Tables A through J are present in this document. (XLS)

S3 File It contains information on websites containing additional supplementary data. (DOCX)
