# Supplementary material for: Comprehensive Map of Molecules Implicated in Obesity
Source: PLoS One. 2016 Feb 17;11(2):e0146759. doi: 10.1371/journal.pone.0146759 (PMC4757102; doi:10.1371/journal.pone.0146759)
Supplement: S1 File — (DOC) [file pone.0146759.s001.doc]

**Table of Contents**

| **Supplementary Files** | | |
| --- | --- | --- |
| **Title** | **Sub-Title** | **Page Numbers** |
| A File | Identification of modules in the network | 5 |
| B File | Quantitative Analysis of Comprehensive Map | 15 |
| C File | Randomisation of Networks | 20 |
| D File | Mapping of Microarray Data to Molecules in Obesity | 27 |
| E File | Relating Side-Effects of Drugs with Comprehensive Map | 29 |
| F File | Comparison of Networks | 33 |
| G File | Text-Mining (TM) Methodology & Tutorial | 35 |
| H File | Details of Annotation Process | 40 |
| I File | Composite Metric Score for Literature Mining | 42 |
| J File | Structure of Map | 48 |
| K File | Description of Docoviz | 51 |
| **Supplementary Figures** | | |
| A Fig. | MCode Prediction. | 6 |
| B Fig. | Modules through Pajek Software. | 7 |
| C Fig. | Power-law distribution of the comprehensive map. | 15 |
| D Fig. | Random Network generated by edge permutation. | 20 |
| E Fig. | Random Network generated by node label permutation. | 21 |
| F Fig. | Node and Edge Randomisation. | 22 |
| G Fig. | Effect of randomisation on clustering coefficient. | 24 |
| H Fig. | Effect of randomisation on mean shortest path. | 25 |
| I Fig. | Relating side effects of sibutramine with molecules. | 32 |
| J Fig. | RefNavigator and its process. | 35 |
| K Fig. | Flow-chart of text-mining approach. | 36 |
| L Fig. | Network Visualization: GraphViz. | 39 |
| M Fig. | Distribution of composite metric score. | 47 |
| N Fig. | Notations used in network. | 49 |
| O Fig. | Obesity network map prepared using Gephi tool. | 50 |
| P Fig. | A schematic diagram depicting work flow of Docoviz. | 52 |
| Q Fig. | Distribution of Docking energies in Positive and Control Dataset (n=23; drug target pairs). | 54 |
|  | **Supplementary Tables** |  |
| A Table | Comparison of modules. | 10 |
| B Table | Cluster prediction by MCode. | 11 |
| C Table | Cluster prediction by GLay. | 12 |
| D Table | Properties of the network and the modules: Network Analyzer & Gephi. | 17-18 |
| E Table | Edge Permutation: In-degree & Out-degree of the original model. | 20 |
| F Table | Effect of number of randomisations (shuffling) on clustering coefficient. | 24 |
| G Table | Randomisations versus Mean Shortest Path. | 24 |
| H Table | GO analysis | 27 |
| I Table | List of representative molecules & their reported (side) effects. | 29-31 |
| J Table | Comparison of Networks. | 33 |
| K Table | Comparison of molecules (Logsdon *et al*, 2012). Also see website. | 34 |
| L Table | List of Dictionaries. | 37 |
| M Table | List of Tenses with examples. | 37 |
| N Table | List of Negations. | 38 |
| O Table | Frequency of occurrences of molecules in obesity abstracts. | 42 |
| P Table | Composite score distribution for genes labelled as hubs in obesity abstracts. | 44 |
| Q Table | Composite score distribution for genes labelled as lesser studied in obesity abstracts. | 45 |
| R Table | Composite score distribution for genes labelled as hubs in obesity network in asthma related abstracts. | 46 |
| S Table | Binding energies. | 53 |
| T Table | Distribution of binding energies in positive and control dataset. | 54 |

Website: <https://sites.google.com/site/obesitynetworkssupp/> for additional supplementary data.

**Mirror Sites: 1**. https://figshare.com/articles/supplementary_Data/2060010

**A File**

**Identification of modules in the network**

Modules are functional building blocks of cellular organisation. Module discovery has been instrumental in elucidating molecular machineries underlying physiological and disease phenotypes. Nevertheless, many challenges confound the interpretation of biological networks and their embedded modular structures. These include difficulties in transformation of thousands of molecular interactions into functionally meaningful models, technological biases and lack of availability of inherent dynamic molecular interaction data under various physiological and environmental conditions. Therefore, the current network models can be best considered as partial and static snapshots of the cell.  To address these issues, experts have recommended the strategy of data integration [1]. Considering this, we used following approaches to identify modules in obesity network (ON):

1. Gene expression based approach.
2. NetCarto tool (Simulated Annealing) based approach.
3. Mcode based approach.
4. Integrated (Knowledge) based approach.
5. **Identification of modules based upon microarray gene expression data:-**

In this approach, we extracted tissue specific gene expression data from gene cards (<http://www.genecards.org/>) and BioGPS for each gene of obesity network (ON). The measurements were obtained for 76 normal human tissues and compartments hybridized against HG-U133A. The Affymetrix MAS5 algorithm was used for array processing and probe sets were averaged per gene. Next, we clustered genes based upon microarray expression data using hierarchical clustering algorithm and observed five major clusters (labelled as modules).

1. M**odule 1**: This module contains 44 genes which show comparatively high expression in brain tissue (S1.xlsx in folder Supplementary File1). Many of these genes (CCK, CPE, and ATP1A1) play major roles in control of hunger and satiation. To illustrate, there are several studies which indicate role of CCK as a satiation factor in humans. CCK levels increase after meals, and infusion of an exogenous CCK-1 receptor agonist, CCK-33, to postprandial levels suppresses food intake [3].
2. **Module 2:** The module contains 101 molecules which show relatively high expression in adipose tissues (S2.xlsx in folder Supplementary File1).
3. **Module 3:** This module contains 201 molecules, majorly expressed in liver tissue (S3.xlsx in folder Supplementary File1).
4. M**odule 4:** The module contains 23 molecules showing relatively high expression in pancreas (S4.xlsx in folder Supplementary File1).
5. **Module 5:** This module contains 63 molecules which show major expression in lungs (S5.xlsx in folder Supplementary File1).


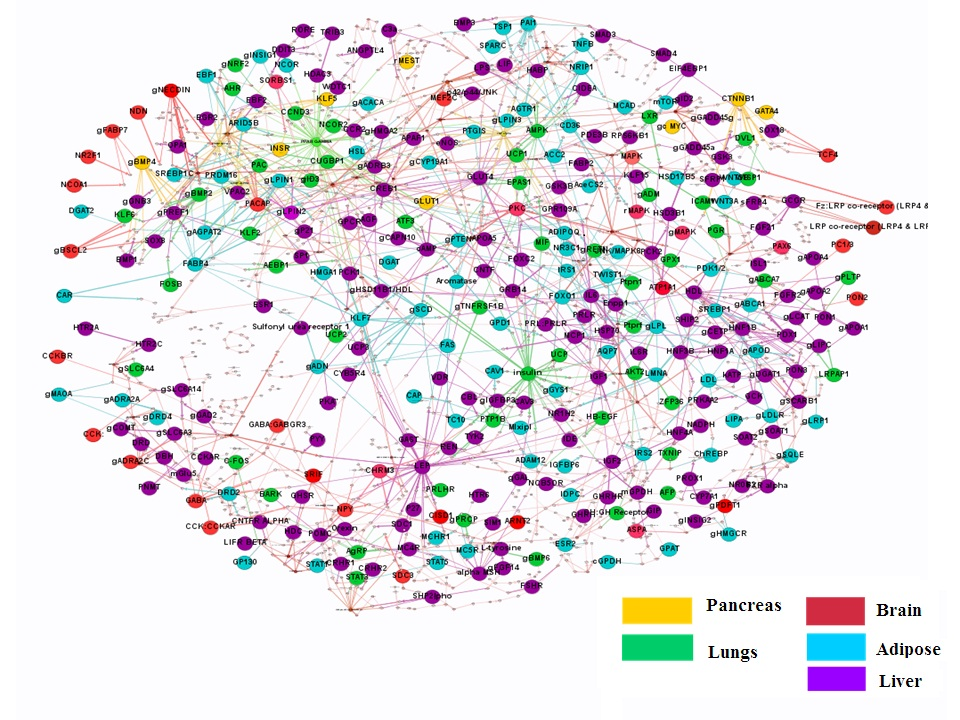


**A Fig.** The obesity network genes are highlighted in different colours based upon tissue specific expression data. For example, red colour circles represent genes showing comparatively higher expression in brain.

1. **Module Generation Based upon Simulated Annealing (NetCarto tool)**

Netcarto identifies modules based upon simulated annealing algorithm (<http://amaral-lab.org/resources/software/netcarto>). Netcarto uses input data in tab separated form and produces six different output files. Using this approach, we identify several module in ON as shown in S2 Fig (S6.xlsx in folder Supplementary File1).
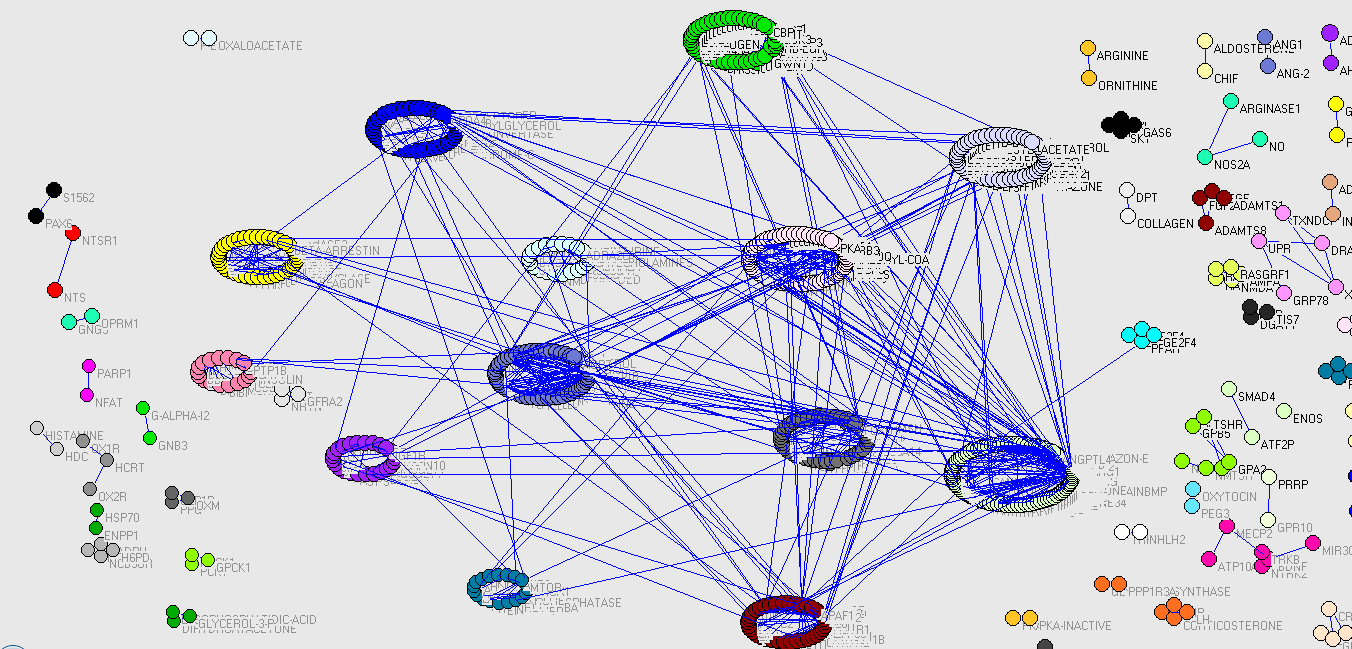


**B Fig. Modules shown in different colours using pajek software.**

**Mcode :-**

   Molecular complex detection “MCode” (a cytoscape plug-in) identifies the highly connected clusters in a network [2]. The algorithm is based on clustering searches for the “seed node” in a network by scoring (score= highest k-core*density) all the nodes based on the density (Density = number of edges/number of possible edges). A parameter named, ‘k-core’, is a part of the graph where every node is connected to other nodes with at least k edges (k=0, 1, 2... n). For every seed node, the algorithm expands the cluster until threshold (score cut-off default is 0.2) is attained and finally post-processes the clusters. For the comprehensive map, we identified 110 clusters (See http://tinyurl.com/au4ho62) “comprehensive map.cys” as an input. The ranking of the clusters are based on the scores i.e. cluster 1 ranks highest (1.65) with 40 nodes and 66 edges. This cluster comprises neuropeptide Y (NPY), pro-opio melanocortin (POMC), agouti-related protein (AgRP), and serotonin. At functional level, they are likely to play role in hunger control. The cluster 2 (34 nodes and 56 edges) includes high and low density lipoprotein (HDL & LDL), lipoprotein lipase (LPL), apolipoprotein (A2 & D), hepatic lipase (LIPC) etc. These molecules are majorly involved in lipid metabolism.

These observations indicate that though each approach produces different results nonetheless, we can ensemble information to reach to consensus. In the following section, we summarise our observations:

1. There are set of genes/molecules termed as hubs which occupy majority of links of network. These include molecules such as leptin (produced mainly by adipocytes in white adipose tissues), insulin (produced by beta cell in pancreas) and PPAR gamma (mostly found in adipose tissue and intestines). Therefore, it is reasonable to conclude that these genes are majorly expressed in distinct tissues (organs) such as brain, liver, pancreas, and adipose tissues.
2. These molecules and their interactions partners can be clustered using physiology or anatomy based schema since there is strong evidence of involvement of molecules and pathways relevant to hunger (brain), adipose tissue & GIT tract including (pancreas & liver).
3. There is a set of molecules which are expressed widely across tissues but still strongly implicated in obesity i.e. Wnt Pathways, insulin, etc.

Hence, we propose the presence of five modules based upon anatomical component, physiological process and predominant hub molecule which can encapsulate most of the information described above. This approach is labelled as knowledge based or integrated approach. After creation of these modules, we also computed the overlap between modules generated through knowledge based approach with automated approaches using Jaccard coefficient.

1. **Integrated Approach**
   1. **Module 1**

This module consists of highly connected nodes which are involved in neuro-hormonal signalling affecting energy homeostasis, hunger and mood, which include leptin, ghrelin and dopamine. Leptin is one of the highly studied molecules in obesity after insulin.  Leptin acts as a satiety factor and its discovery has paved the way for the study of adipocyte derived factor in energy balance homeostasis. Further, the secretion of the leptin is directly proportional to amount of fat cells [4]. Recently, leptin replacement therapy has been proposed to treat obese individuals [5]. Frequent association of obesity with clinical depression can be explained by the impaired leptin activity in brain [6].

              Ghrelin act as an endogenous ligand for growth hormone secretagogue receptor (GHSR). It has been reported to be involved in energy regulation and appetite signalling through activation of peptides, including AgRP, NPY and POMC [7]. Rise and fall in plasma ghrelin levels before and after food intake supports the hypothesis that ghrelin plays a physiological role in meal initiation in humans [8]. Ghrelin levels are altered in individuals suffering from Prader-Willi and Cushing's syndrome [9]. A meta-analysis linked gastrointestinal hormones, ghrelin and obestatin levels with obesity [10].

               The importance of dopamine signaling in obesity has been demonstrated by the alteration of dopamine receptor levels with changes in body mass index (BMI) [10]. Apart from these, several other molecules have been reported in context of obesity; therefore, we have described their roles at our website: <http://tinyurl.com/kazahj6>.

- 1. **Module 2**

Obesity is a major risk factor for non-insulin dependent diabetes mellitus (NIDDM) [10]. Insulin is a central molecule in pathophysiology of type 2 diabetes and also appears in large number of abstracts related to obesity (23,164 abstracts; 27% of total dataset) in humans. Module 2 primarily encapsulate insulin and its interactions with other molecules, for instance, apolipoprotein A-V (APOA5), forkhead box C2 (FOXC2), macrophage migration inhibitory factor (MIF), uncoupling protein (UCP) and v-akt murine thymoma viral oncogene homolog 2 (AKT2). This module builds a link between tightly coupled clinical conditions- obesity and type 2 diabetes.

- 1. **Module 3**

Lipid storage and metabolism is affected frequently in obese patients leading to dyslipidemia, exposing them to cardiovascular risks [12] and atherosclerosis [13]. The third module maps interactions, catalysis and processing of molecules involved in lipid metabolism, including acetyl CoA, aspartate, mevalonate, cholesterol, cholic acid, and diacylglycerol.

- 1. **Module 4**

It is the largest module in the network and majorly consists of transcription factors involved in adipose tissue differentiation and other biological activities in humans. The interactions are dominated by molecules like peroxisome proliferator-activated receptors-PPAR (α, β, γ) and CCAAT/enhancer-binding proteins-C/EBP (α, β, γ).  The molecules such as PPAR γ (with 41 edges) provide indirect connections with lesser studied genes/molecules reported in context of obesity. This module is categorised into another sub-module labelled as “4A” to incorporate set of molecules distinct from transcription factors.

- 1. **Module 4A**

Though the Wnt pathway has been shown to play a major role in embryogenesis and some of the cancers, it has also emerged as an important regulator of adipocyte differentiation [14]. In addition, recent evidence of obesity treatment using traditional herbal medicine, SH21B, has indicated about anti-adipogenic mechanism mediated by Wnt-β catenin signalling [15].

- 1. **Module 5**

The last module contains information about disjoint set of genes/proteins involved in obesity which are difficult to categorize due to inadequate information.

**Comparison of modules generated from different approaches:**

We used Jaccard-index J = |A∩B|/|AUB| to compare modules generated from different approaches and the results are enclosed (See Supplementary File 1 folder at website). A representative data is enclosed below (Table1.xlsx in Supplementary File 1 folder at website).

**A Table.** Comparison of modules generated from integrated approach and NetCarto based methods. We compute Jaccard Index as shown below. The information in parenthesis is number of molecules in each dataset. We highlight best matches in yellow colour.

| **Integrated Vs NetCarto (Modules)** | **Module5 (62)** | **Module14 (38)** | **Module21 (48)** | **Module27 (50)** | **Module31 (22)** | **Module33 (49)** |
| --- | --- | --- | --- | --- | --- | --- |
| **Module1 (129)** | **0.021** | **0** | **0.017** | **0** | **0.153** | **0.203** |
| **Module2 (158)** | **0.005** | **0.043** | **0.04** | **0.015** | **0** | **0.062** |
| **Module3 (71)** | **0** | **0.048** | **0** | **0.315** | **0** | **0** |
| **Module4 (272)** | **0.201** | **0.084** | **0.016** | **0.07** | **0.007** | **0.003** |
| **Module5 (40)** | **0.01** | **0** | **0.492** | **0.011** | **0** | **0** |

**MCODE modules Vs Modules generated through integrated approach:**

We also computed the overlap percentage of these cluster generated from MCODE with the respective modules. For instance, cluster 2 shows the highest overlap with module 2 (21.9%) whereas cluster 40 has the least overlap with module 2 (1.6 %).

**B Table.** Display the data generated by MCode. The columns shows information for each cluster i.e. score, number of constituent nodes within the clusters (labelled as A) and edges. The nodes of these clusters are mapped to the modules generated from integrated approach (labelled as set B). The percentage overlap of these clusters to their respective modules [(A/B)*100] are computed. We show data for top 14 clusters for representative purposes.

| **Cluster from Mcode** | **Score** | **Nodes**  **(A)** | **Edges** | **Modules generated from integrated approach** | **Molecules in module from integrated approach (B)** | **% Overlap**  **(A/B)*100** |
| --- | --- | --- | --- | --- | --- | --- |
| 1 | 1.65 | 40 | 66 | Module 1 | 288 | 13.88 |
| 2 | 1.647 | 34 | 56 | Module 3 | 155 | 21.93 |
| 3 | 1.625 | 8 | 13 | Module 4a | 83 | 9.63 |
| 4 | 1.583 | 12 | 19 | Module 2 | 368 | 3.26 |
| 5 | 1.567 | 30 | 47 | Module 4 | 631 | 4.75 |
| 6 | 1.545 | 11 | 17 | Module 4 | 631 | 1.74 |
| 7 | 1.545 | 11 | 17 | Module 4a | 83 | 13.25 |
| 8 | 1.545 | 11 | 17 | Module 1 | 288 | 3.81 |
| 9 | 1.522 | 23 | 35 | Module 4 | 631 | 3.64 |
| 10 | 1.5 | 12 | 18 | Module 4 | 631 | 1.90 |
| 11 | 1.5 | 8 | 12 | Module 2 | 368 | 2.17 |
| 12 | 1.455 | 11 | 16 | Module 2 | 368 | 2.98 |
| 13 | 1.429 | 7 | 10 | Module 5 | 196 | 3.57 |
| 14 | 1.4 | 5 | 7 | Module 5 | 196 | 2.55 |

**Fast Greedy Algorithms:**

The algorithm considers each node as an individual community and extends the size of the node with the neighbours until the maximum modularity is attained leading to formation of a cluster [16]. GLay, a cytoscape plug-in [17] was used to run algorithm and 60 clusters were identified with a modularity score of 0.85 using input file “comprehensive map.cys”. The overlap percentage is computed for the clusters having size >60. We observed that cluster 9 show overlap of 100 percent with module 4a (See http://tinyurl.com/pgchu6x).

**C Table.** Cluster prediction by GLay.

| **S.No.** | **Cluster ID** | **Cluster Size: No. of Nodes**  **(A)** | **Edges** | **Match with Comprehensive**  **Map: Module** | **Molecules in Module**  **(B)** | **% Overlap (A/B)*100** |
| --- | --- | --- | --- | --- | --- | --- |
| 1 | 5 | 146 | 281 | Module 4 | 631 | 23.13 |
| 2 | 3 | 110 | 187 | Module 4 | 631 | 15.84 |
| 3 | 19 | 95 | 148 | Module 1 | 288 | 32.98 |
| 4 | 9 | 87 | 134 | Module 4a | 83 | 100 |
|  |  |  |  | Module 1 | 288 | 1.04 |
| 5 | 21 | 84 | 112 | Module 2 | 368 | 22.82 |
| 6 | 4 | 81 | 116 | Module 3 | 155 | 52.25 |
| 7 | 1 | 79 | 103 | Module 5 | 196 | 12.75 |
|  |  |  |  | Module 4 | 631 | 8.39 |
| 8 | 7 | 70 | 105 | Module 4 | 631 | 11.09 |
| 9 | 13 | 67 | 104 | Module 2 | 368 | 18.20 |
| 10 | 10 | 65 | 92 | Module 4 | 631 | 10.30 |

**References**

1. Mitra K, Carvunis A-R, Ramesh SK, Ideker T. Integrative approaches for finding modular structure in biological networks. Nat Rev Genet. 2013; 14: 719–732.
2. Little T, Horowitz M, Bisser C. Role of cholecystokinin in appetite control and body weight regulation. Obesity Reviews. 2005; 6: 297–306.
3. Bader JS. Greedily building protein networks with confidence. Bioinformatics. 2003; 19:1869-1874.
4. Fruhbeck G. Intracellular signalling pathways activated by leptin. Biochem J. 2006; 393: 7–20.
5. Yamada N, Katsuura G, Ochi Y, Ebihara K, Kusakabe T, et al. Impaired CNS leptin action is implicated in depression associated with obesity. Endocrinology. 2011; 152: 2634-2643.
6. Toshinai K, Date Y, Murakami N, Shimada M, Mondal MS, et al. Ghrelin-Induced Food Intake Is Mediated via the Orexin Pathway. Endocrinology. 2003; 144: 1506-1512.
7. Nogueiras R, Tschop MH, Zigman JM. Central nervous system regulation of energy metabolism: ghrelin versus leptin. Ann. N. Y. Acad. Sci. 2008; 1126: 14-19.
8. Scerif M, Goldstone AP, Korbonits M. Ghrelin in obesity and endocrine diseases. Mol. Cell. Endocrinol. 2011; 340: 15-25.
9. Zhang N, Yuan C, Li Z, Li J, Li X, et al. Meta-Analysis of the Relationship between Obestatin and Ghrelin Levels and the Ghrelin/Obestatin Ratio With Respect to Obesity. American Journal of the Medical Sciences. 2011; 341: 48-5.
10. KD, Niswender Daws LC, Avison MJ, Galli A. Insulin Regulation of Monoamine Signaling: Pathway to Obesity. Neuropsychopharmacology. 2011; 61: 1123-8.
11. Gallagher EJ, LeRoith D. Insulin, insulin resistance, obesity, and cancer. [Current Diabetes Reports](http://link.springer.com/journal/11892). 2010; 10: 93-100.
12. Toh SA, Levin M, Rader DJ. Atherogenic Lipid Metabolism in Obesity. Met. basis obes. 2011; 293-309.
13. Purnell JQ., Hokanson JE, Cleary PA, Nathan,DM, Lachin JM, et al. The Effect of Excess Weight Gain with Intensive Diabetes Treatment on Cardiovascular Disease Risk Factors and Atherosclerosis in Type 1 Diabetes: Results from the Diabetes Control and Complications Trial / Epidemiology of Diabetes Interventions and Complications Study [DCCT/EDIC] Study. Circulation. 2012; 10.1161.
14. Prestwich TC, Macdougald OA. Wnt/β-catenin signaling in adipogenesis and metabolism. Current Opinion in Cell Biology. 2007; 19: 612-617.
15. Lee H, Bae S, Yoon Y. WNT/β-catenin pathway mediates the anti-adipogenic mechanism of SH21B, a traditional herbal medicine for the treatment of obesity. [Journal of Ethnopharmacology](http://www.sciencedirect.com/science/journal/03788741). 2011; 133: 788-795.
16. Clauset A, Newman M, Moore C. Finding community structure in very large networks. Physical Review E. 2004; 70.066111.

**B File**

**Quantitative Analysis of Comprehensive Map**

Many real world networks such as internet, biological networks and social networks show distinct topological properties and architecture. To understand the properties of constructed obesity network, we analysed several topological parameters as described below.

A) **The degree distribution parameter**, P(*k*), gives the probability that a randomly selected node has exactly *k*links. The shape of the degree distribution is different in random, scale-free and hierarchical networks [1]. The scale-free nature of the network is predicted from the degree distribution which approximates a power law given by *P*(*k*) ~ *k*^–γ^, where γ is the degree exponent which determines the property of the system such as allowing presence of few nodes with very large degrees to exist in the network. The degree exponent of various real world network, having scale-free tendency, are within the range of 2 < γ ≤ 3 and for biological networks- γ is between 2.0-2.4 [2].  Since our network is a directed graph, we computed in-degree and out-degree for nodes separately. In our case, the histogram of connectivities can be fitted by a power law with in-degree parameter (γ_in_) as 2.19 for and out-degree parameter (γ_out_) as 2.11 suggesting scale free behaviour (C Fig). The degree distributions for constituent modules were also determined (See D Table).

**
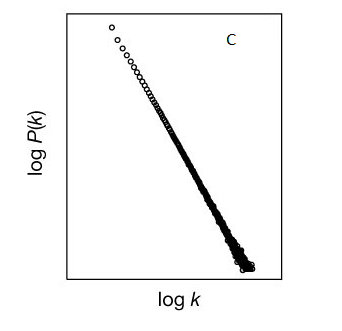

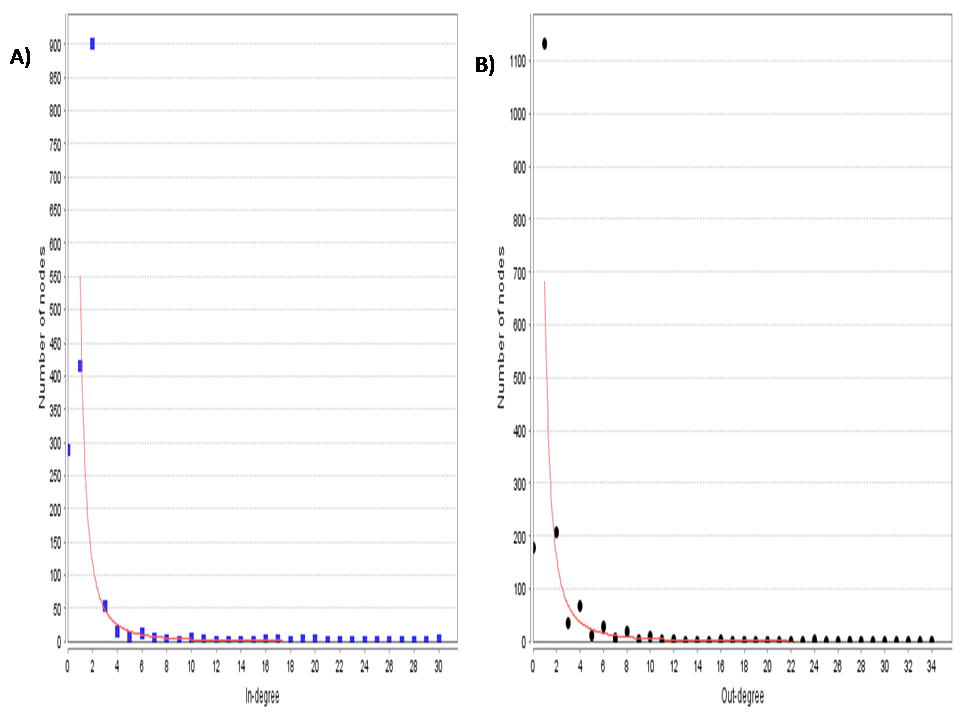
**

**C Fig.** The network connectivity can be characterized by the probability, *P*(*k*), that a node has *k* links. *P*(*k*) for a scale-free network has no well-defined peak, and for large *k* it decays as a power-law, *P*(*k*) 
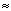
 1/*k*
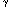
, appearing as a straight line with slope -
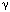
 on a log–log plot (See C). (A) In-Degree Distribution. (B) Out-Degree Distribution. In-degree/out-degrees (k) are represented on X axis and number of nodes are shown on Y axis.

   B) **Clustering Coefficient-** If node A of a network is connected to B, and B is connected to C, then it is highly probable that A also has a direct link to C. This phenomenon can be quantified using the clustering coefficient. It calculates the average local neighbourhood in a network [3] and varies considerably across the network [4]. If the value of clustering co-efficient is close to 0, then it is interpreted as most of the nodes in the network have less than two neighbours- implying tree-like structure [5].  Our comprehensive map and the modules show clustering coefficient value close to 0**.**Another important measure of the network’s structure is average clustering coefficient *C*(*k*), which suggests the modularity of the metabolic network [6]. Using various tools such as Network analyzer and Gephi, we found average clustering coefficient for our network to be 0, suggesting presence of tree like structure.

C) **Network diameter**is the maximum distance between two nodes. If the network is disconnected then diameter is calculated by the average of maximum distances between connected components. We observed network diameter for ON as 46.

D) **The average shortest path length** is measured as an average number of edges separating any two nodes in the network. Our network consisting of 1788 nodes shows average shortest path length of 15.85. This result support scale free nature of ON [7].

E) **The average number of neighbours** indicates the average connectivity of a node in the network. We found this parameter within the range of 2.2 to 2.4 for our network and its constituent modules.

F) **Network Density** determines the compactness, which depends on the isolated nodes in the network. A network having isolated nodes will have density of 0 [8].

**D Table.** display the properties of the network and its constituent modules computed using Network Analyzer and Gephi tool.

1. **Network Analyzer**

| **Properties** | | **Comprehensive Map** | **Module 1** | **Module 2** | **Module 3** | **Module 4** | **Module 4a** | **Module 5** |
| --- | --- | --- | --- | --- | --- | --- | --- | --- |
| Nodes | | 1788* | 288 | 368 | 155 | 631 | 83 | 196 |
|  | In-degree |  |  |  |  |  |  |  |
|  | Γ | 2.192 | 2.282 | 2.234 | 2.147 | 1.88 | 2.176 | 2.928 |
|  | R^2^ | 0.858 | 0.839 | 0.786 | 0.805 | 0.768 | 0.076 | 0.621 |
| Degree Distribution | Out-degree |  |  |  |  |  |  |  |
|  |  |  |  |  |  |  |  |  |
|  | Γ | 2.11 | 2.233 | 1.771 | 2.235 | 1.841 | 1.393 | 2.386 |
|  | R^2^ | 0.913 | 0.942 | 0.756 | 0.907 | 0.826 | 0.749 | 0.697 |
| Clustering Co-efficient | | 0 | 0 | 0 | 0 | 0 | 0 | 0 |
| Connected Components | | 34 | 2 | 3 | 3 | 4 | 1 | 35 |
| Diameter | | 46 | 23 | 23 | 23 | 40 | 18 | 8 |
| Average Shortest Path-length | | 15.85 | 8.5 | 8.3 | 9.8 | 13.3 | 7.5 | 2.4 |
| Average number of neighbours | | 2.37 | 2.28 | 2.23 | 2.32 | 2.46 | 2.21 | 1.72 |
| Network Density | | 0 | 0 | 0 | 0 | 0 | 0 | 0 |

γ - Power law exponent, R2 -Value for power law fit. *- The actual number of nodes is 804. Since, Cytoscape consider each reaction as a node therefore number has been increased to 1788.

**(ii) Gephi**

| **Properties** | **Comprehensive Map** | **Module 1** | **Module2** | **Module 3** | **Module 4** | **Module 4a** | **Module 5** |
| --- | --- | --- | --- | --- | --- | --- | --- |
| **Nodes** | 1788 | 288 | 368 | 155 | 631 | 83 | 196 |
| **Edges** | 2131 | 335 | 412 | 181 | 776 | 92 | 171 |
| **Average Degree** | 1.192 | 1.163 | 1.12 | 1.168 | 1.23 | 1.1 | 0.872 |
| **Average Weighted Degree** | 1.604 | 1.465 | 1.5 | 1.471 | 1.74 | 1.566 | 1.117 |
| **Network Diameter** | 46 | 23 | 23 | 23 | 40 | 18 | 8 |
| **Density** | 0.001 | 0.004 | 0.003 | 0.008 | 0.002 | 0.014 | 0.004 |
| **Modularity** | 0.877 | 0.833 | 0.845 | 0.77 | 0.832 | 0.758 | 0.952 |
| **No. of Clusters** | 62 | 19 | 21 | 14 | 26 | 9 | 32 |
| **Connected Components** | |  |  |  |  |  |  |
| **Weakly Connected** | 34 | 2 | 3 | 3 | 4 | 1 | 35 |
| **Strongly Connected** | 1389 | 361 | 361 | 119 | 525 | 66 | 194 |
| **Average Clustering co-efficient** | 0 | 0 | 0 | 0 | 0 | 0 | 0 |
| **Average Path length** | 15.855 | 8.505 | 8.372 | 9.859 | 13.37 | 7.578 | 2.4 |

**References**

1. Barabasi AL, Oltvai ZN. Network Biology: understanding the cell’s functional organization. Nature Reviews Genetics. 2004; 5:101-113.
2. Goh K,Oh E,Jeong H, Kahng B, Kim D. Classification of scale-free networks. PNAS. 2002; 99:12583–12588..
3. Watts DJ, Strogatz SH.Collective dynamics of 'small-world' networks. Nature.1998; 393:440-442.
4. DeSilva E, Stumpf M. Complex networks and simple models in biology. J. R. Soc. Interface .2005; 2:419–430.
5. Ravasz E,Somera AL,Mongru DA,Oltvai ZN,Barabasi AL. Hierarchical Organization of Modularity in Metabolic Networks.Science. 2002; 297:1551-1555.
6. Barrat A, Barthelemy M, Pastor R, Vespignani A. The architecture of complex weighted networks. PNAS. 2004; 0400087101.
7. Radrich K, Tsuruoka Y, Dobson P, Gevorgyan A, Swainston N, Baart G, Schwartz J. Integration of metabolic databases for the reconstruction of genome-scale metabolic networks. BMC Systems Biology. 2010; 4:114.
8. Ravasz E,Somera AL,Mongru DA,Oltvai ZN,Barabasi AL. Hierarchical Organization of Modularity in Metabolic Networks.Science. 2002; 297:1551-1555.

**C File**

**Randomisation of Networks**

***Null Model 1: Edge randomisation***

In this approach, the edges are shuffled by keeping the number of edges to a node i.e. degree of node (in-degree and out-degree) as same as that of nodes of original map (ON). Consider a representative network with seven nodes (A, B, C, D, E, F and G) and their edges labelled as E_BA_, E_AD_ etc. (D Fig (i) and D Table). The edges of original network are shuffled so that edge labelled as E_BA_ becomes E_AG_ [D Fig (ii)].

**E Table:** depicts the in-degree and out-degree for the original map in D Fig.

|  | In-degree | Out-degree |
| --- | --- | --- |
| **A** | 1 | 2 |
| **B** | 0 | 1 |
| **C** | 1 | 1 |
| **D** | 2 | 0 |
| **E** | 2 | 1 |
| **F** | 1 | 1 |
| **G** | 2 | 1 |


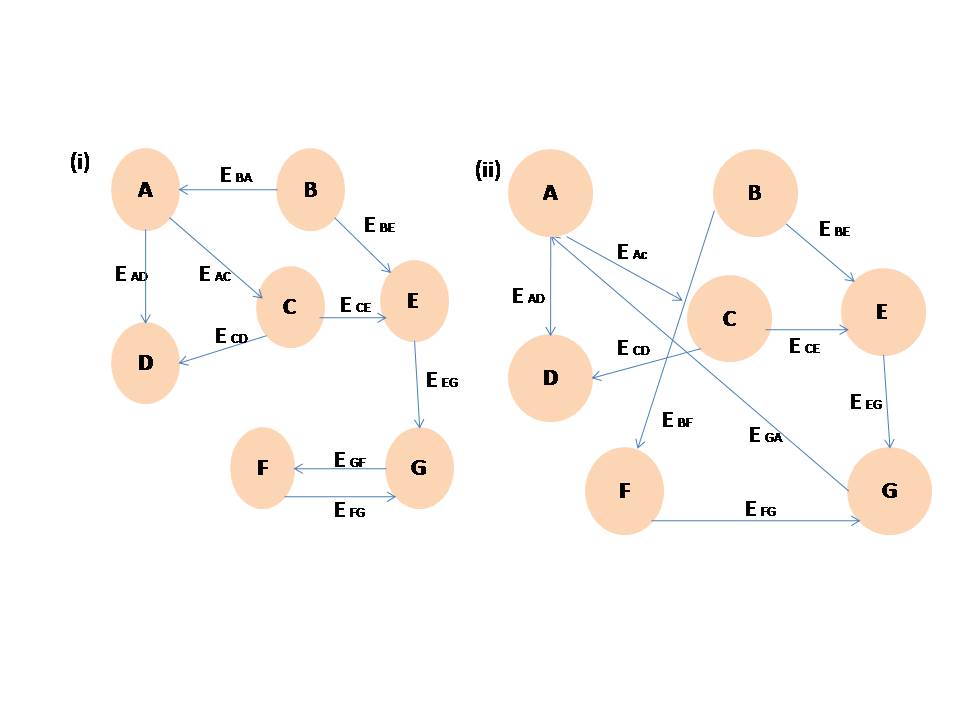


**D Fig. (i)** Original network and **(ii)** Random model (After edge permutations).

We used network analyzer tool (NeAT) [1] to implement this strategy. In this randomization, the degree of each node is preserved. (Detailed procedures as well as datasets are available at “http://tinyurl.com/ad6c9ya**”**).

***Null Model 2: Node randomisation***:

In this model, the label of the node is permuted keeping the global degree distribution preserved. This leads to changes in degree of individual nodes [2]. In a sample network (E Fig), the labels of the nodes are shuffled using perl scripts. For example, ‘A’ node of the original model is replaced with the node labelled as ‘B’ in random model. Similarly, node ‘B’ (original model) was replaced with node ‘E’.
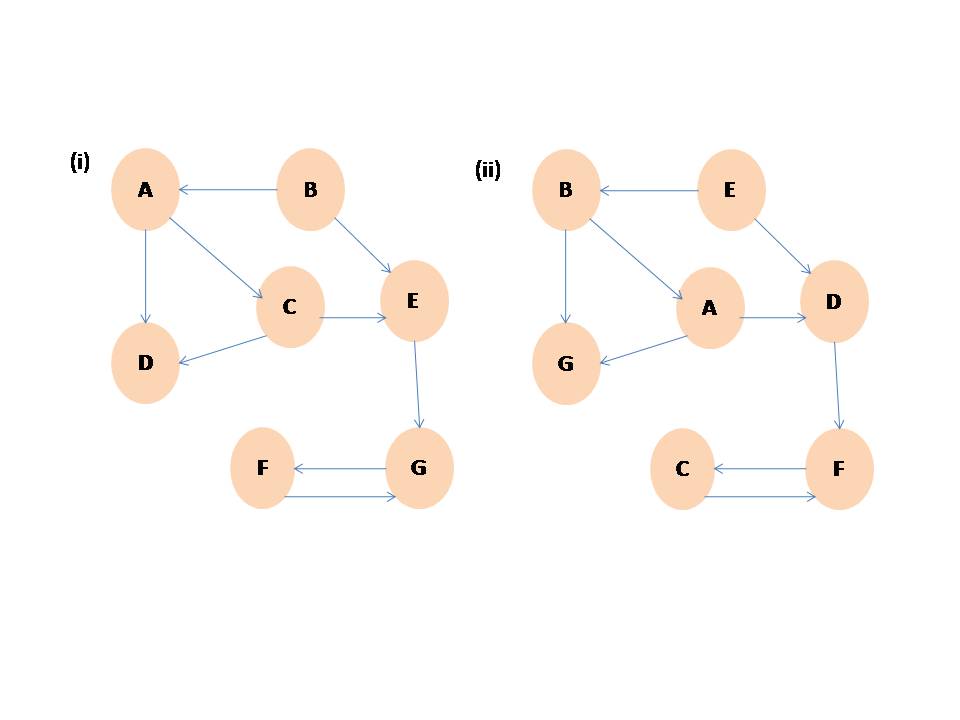


**E Fig.** Random Network generated by node label permutation (i) Original model (ii) Random model (After node label permutation).

***Null Model 3: Node and edge randomisation***

Here, we combine both the approaches (1 and 2) to generate random graphs where both nodes and edges are shuffled.


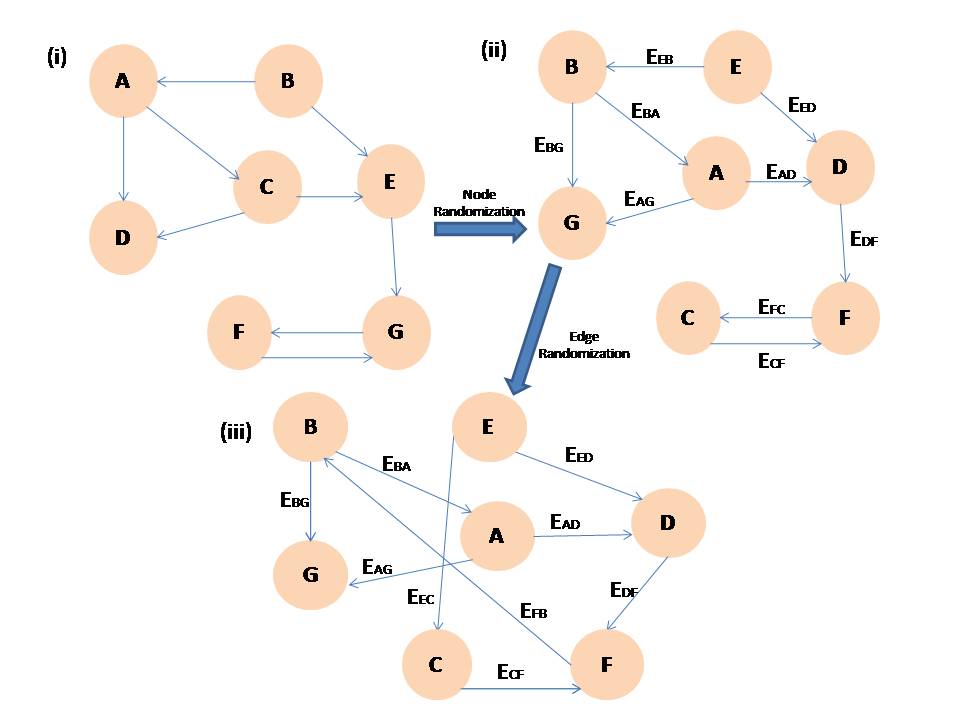


**F Fig.** Node and Edge Randomisation. (i) Original model (ii) Node label shuffled model (iii) Edge shuffled model

In total 4 random models (labelled as 1, 2, 3 and 4) corresponding to each type of randomisation were generated. We compared properties of randomly generated network with original network (See H Table in S2 File).

***Null Models 4:***

     Here, we generated random models using classical random network theory [3]. In Watts-Strogatz model, the random graph is produced with small-world properties, including short average path length and high clustering. In Barabasi-Albert model, the generation of random graph is based on the connected seed network of s nodes. Every other node (n-s) is added one at a time, and initially connected to m existing nodes. The resulting random network has a power-law degree distribution with a scale-free nature. To see the effect of number of nodes on topological properties, we constructed random networks consisting 100, 1000 and 1799 nodes and their properties were computed (See H Table in S2 File).

**Effect of Randomisation on Network Properties:**

We also generated randomized networks using random network module of cytoscape [4]. The obesity network (true) exhibit different properties when compared to 19 randomized networks (controls) obtained by shuffling the obesity network associations while keeping the degree distribution of nodes fixed (E Table). We find that clustering coefficient increases from 0 (in true network) to 0.002212 (randomized network with 30000 shuffling. See G Fig & F Table). This pattern is reversed in case of mean shortest path, which reduced from 18 to 11 units (See G Table and H Fig).

**F Table.** Effect of number of randomisations (shuffling) on clustering coefficient.

| **Number of Randomisations** | **Clustering Coefficient** |
| --- | --- |
| 10 | 1.81E-05 |
| 100 | 2.24E-04 |
| 200 | 3.19E-04 |
| 500 | 7.05E-04 |
| 1000 | 9.50E-04 |
| 2000 | 0.0011257 |
| 3000 | 0.001308 |
| 4000 | 0.0014183 |
| 5000 | 0.001692 |
| 8000 | 0.0015332 |
| 10000 | 0.0016502 |
| 12000 | 0.0018381 |
| 15000 | 0.0019015 |
| 18000 | 0.0021075 |
| 20000 | 0.0019195 |
| 22000 | 0.0021552 |
| 25000 | 0.0021873 |
| 28000 | 0.0021985 |
| 30000 | 0.0022129 |


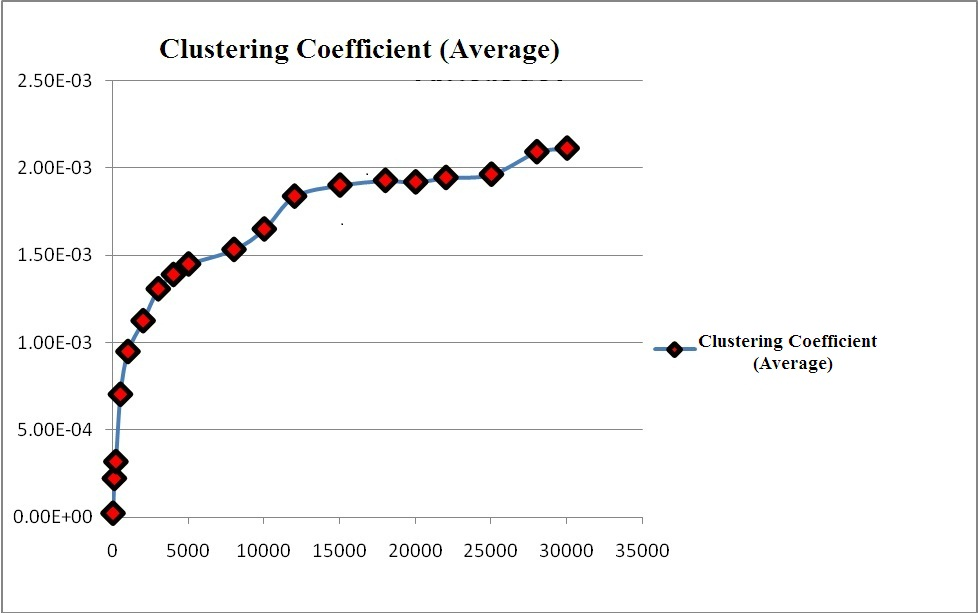


**G Fig.** Effect of randomisations (shuffling) on clustering coefficient. X axis represent the number of randomisations and Y axis represent the clustering coefficient.

**G Table.** Randomisations versus Mean Shortest Path.

| **Number of Randomisations** | **Mean Shortest Path** |
| --- | --- |
| 10 | 15.79 |
| 100 | 14.68 |
| 200 | 14.35 |
| 500 | 13.27 |
| 1000 | 12.79 |
| 2000 | 12.27 |
| 3000 | 12.31 |
| 4000 | 12.04 |
| 5000 | 12.01 |
| 8000 | 11.70 |
| 10000 | 11.59 |
| 12000 | 11.58 |
| 15000 | 11.63 |
| 18000 | 11.44 |
| 20000 | 11.66 |
| 22000 | 11.52 |
| 25000 | 11.61 |
| 28000 | 11.31 |
| 30000 | 11.39 |


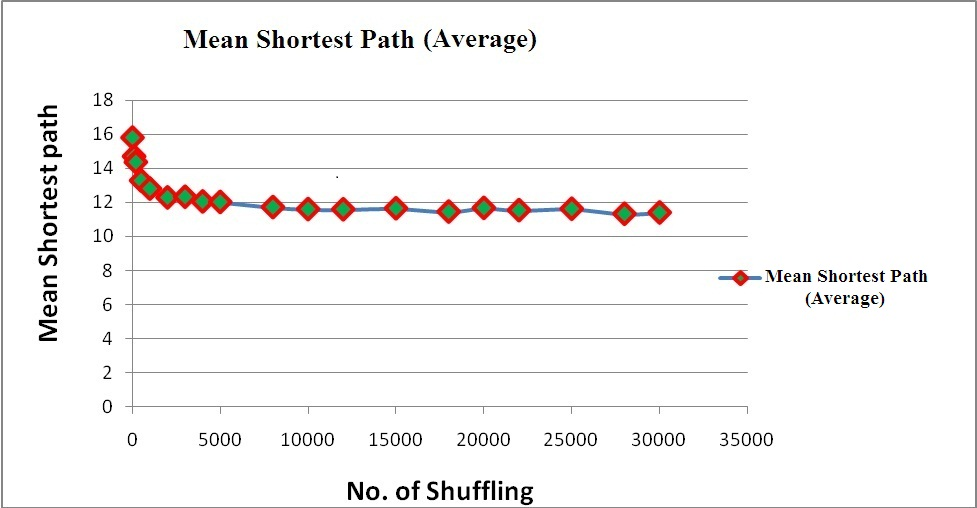


**H Fig.** Effect of randomisations (shuffling) on mean shortest path. X axis represent the number of randomisations and Y axis represent the mean shortest path.

**References**

1. Brohee, S., Faust, K., Lima-Mendez, G., Vanderstocken, G., & van Helden, J. Network Analysis Tools: from biological networks to clusters and pathways. Nature protocols. 2008; 3(10): 1616-1629.
2. Yang, Y., Dong, Y., & Nitesh, V.C. Predicting Node Degree Centrality with the Node Prominence Profile. Scientific Reports. 2014; 4: 7236.
3. Erdos P., Renyi A. On the evolution of random graphs. Publ. Math. Inst. Hung. Acad. Sci. 1960; 5:17–61.
4. Shannon P., Markiel A., Ozier O.,Baliga NS., Wang JT., Ramage D.,Amin N.,Schwikowski B.,Ideker T. Cytoscape: A Software Environment for Integrated Models of Biomolecular Interaction Networks. [Cold Spring Harbor Laboratory Press](http://genome.cshlp.org/site/misc/terms.xhtml). 2003; 13:2498-2504.

**D File**

**Mapping of Microarray Data to Molecules in Obesity**

The microarray data was obtained from the Gene Expression Atlas database (<http://www-test.ebi.ac.uk/gxa/>) in context of obesity. Thereafter, we searched molecules obtained from DC and TM approaches in the microarray dataset. Next, they were labelled as up regulated, down regulated, up/down and showing no change in expression. In addition, we did not find expression data for large number of molecules from this resource. We conducted gene ontology analysis for the above mentioned groups using Bingo [1]. In up-regulated class (DC group), majority of molecules are classified under “protein binding” group (p-value = 1.68E-03). The molecules belonging to TM group (BMP2, ATF3, TP53 etc.) were mapped to “protein dimerization activity” (p-value = 1.90E-09) (See S7 Table at website).

**H Table.** Provides details about GO analysis of the molecules implicated in obesity. The molecules were divided into 3 classes based upon their expression profile obtained from gene expression atlas. They were classified into up-regulated, down-regulated and non-differentially expressed. Next, molecules were mapped into three functional categories namely cellular compartment (CC), molecular function (MF) and biological processes (BP) and their sub-categories.

**Application of Gene Expression Omnibus (GEO)**

In the above mentioned experiment, we observed that many of the important genes such as leptin and insulin were found to show no change in expression. In many cases, data pertaining to these genes was absent. To find out the reason, we used gene expression omnibus profiles database for the list of genes in ON. For example, when we searched leptin and obesity for up/down regulated genes as a filter in GEO profiles, we retrieved 3069 hits representing several gene expression studies. During manual screening, we found most of them were not relevant. Next, we used ‘Lep and Obesity’ as a query with a filter of up/down regulated genes which generated nine hits. Out of these 9 studies, we found only two studies can be used to determine whether leptin is differentially expressed in obesity. We labelled leptin as up-regulated in obesity condition based upon information retrieved from GEO profiles. This information was not found when we used Gene Expression Atlas database. Using similar approach for rest of the genes in GEO, we find that out of 1,268 obesity network genes (obtained from TM approach): 34.5 % are up regulated and 27.58 % are down regulated (See GEO.xlsx at website).

**References**

1. Maere S, Heymans K, Kuiper M. BiNGO: a Cytoscape plugin to assess overrepresentation of Gene Ontology categories in Biological Networks. Bioinformatics Applications Note. 2005; 16:3448–3449.

**E File**

**Relating Side-Effects of Drugs with Comprehensive Map**

A drug molecule not only targets its desired protein but also targets other proteins which are known as off-targets of that drug [1]. These unpredicted binding targets in some cases, mediates the physiological effect of a drug, even if the drug is designed specifically to target one particular protein. In addition, drug side effects can be attributed to the number of reasons such as downstream pathway perturbations, kinetic and dosage effects, drug-drug interferences, insufficient metabolization, aggregation or irreversible binding to primary targets. It has been accepted that drug-protein binding interaction is the primary step in triggering molecular events in the biological system when a drug is administered. Identification of these unexpected drug-protein interactions could lead to discovering new therapeutic targets. For instance, sildenafil was developed to treat angina, but its side effects of prolonged penile erections led to change in the therapeutic area of drug [2]. There are many drugs which were discovered for obesity treatment but withdrawn due to their adverse effects. We attempted to address these issues by studying side-effects and therapeutics of the FDA approved drug for obesity known as orlistat. The drug molecules and their associated-effects were obtained from SIDER database [3]. We also used Ingenuity knowledgebase for genes (protein targets), and their potential role in side-effects because of interactions with drugs. We obtained a list of 46 molecules from comprehensive map along with their associated effects from SIDER. Some of these molecules had more than one effect. For example, peroxisome proliferator activator receptor gamma (PPARG) is associated with lipodystrophy, obesity and type 2 diabetes (See I Table).

I **Table.** Shows the list of representative molecules & their reported (side) effects. The molecules and their effects are supported with literature evidence.

| **Gene Symbol** | **Side-effect** | **Evidence** |
| --- | --- | --- |
| ACE | Angioedema | <http://www.ncbi.nlm.nih.gov/pubmed/9530537> |
| ACE | Atherosclerotic vascular disease | <http://www.ncbi.nlm.nih.gov/pubmed/16146835> |
| ACE | Cough | <http://www.ncbi.nlm.nih.gov/pubmed/18405793> |
| ACE | Hyperkalemia | <http://www.ncbi.nlm.nih.gov/pubmed/8828013> |
| ACE | Hypernatremia | <http://www.ncbi.nlm.nih.gov/pubmed/2220796> |
| ACE | Hypotension | <http://www.ncbi.nlm.nih.gov/pubmed/10464907> |
| ATP1A2 | Migraine | <http://www.ncbi.nlm.nih.gov/pubmed/16508935> |
| BDNF | Diarrhoea | <http://www.ncbi.nlm.nih.gov/pubmed/?term=12403983> |
| COMT | bipolar_disorder | <http://www.ncbi.nlm.nih.gov/pubmed/15211633> |
| COMT | Schizophrenia | <http://www.ncbi.nlm.nih.gov/pubmed/16476412> |
| COMT | Nausea | <http://www.ncbi.nlm.nih.gov/pubmed/21570824> |
| COMT | Vomiting | <http://www.ncbi.nlm.nih.gov/pubmed/21570824> |
| CYP19A1 | Flushing | <http://www.ncbi.nlm.nih.gov/pubmed/16343926> |
| CYP19A1 | hot_flashes | <http://www.ncbi.nlm.nih.gov/pubmed/15494636> |
| CYP19A1 | Osteoporosis | <http://www.ncbi.nlm.nih.gov/pubmed/10750565> |
| CYP19A1 | Virilisation | <http://www.ncbi.nlm.nih.gov/pubmed/13678849> |
| DRD2 | Nausea | <http://www.ncbi.nlm.nih.gov/pubmed/21570824> |
| DRD2 | Vomiting | <http://www.ncbi.nlm.nih.gov/pubmed/21570824> |
| DRD2 | Akathisia | <http://www.ncbi.nlm.nih.gov/pubmed/21406165> |
| ENPP1 | type_2_diabetes | <http://www.ncbi.nlm.nih.gov/pubmed/19017751> |
| ESR1 | Osteoporosis | <http://www.ncbi.nlm.nih.gov/pubmed/11846326> |
| ESR1 | Syncope | <http://www.ncbi.nlm.nih.gov/pubmed/11113008> |
| HTR2A | Anxiety | <http://www.ncbi.nlm.nih.gov/pubmed/16873667> |
| HTR2A | bipolar_disorder | <http://www.ncbi.nlm.nih.gov/pubmed/10893484> |
| HTR2A | obsessive_compulsive_disorder | <http://www.ncbi.nlm.nih.gov/pubmed/18339223> |
| HTR2A | Schizophrenia | <http://www.ncbi.nlm.nih.gov/pubmed/9264136> |
| HTR2A | Tremor | <http://www.ncbi.nlm.nih.gov/pubmed/7675955> |
| HTR2A | Akathisia | <http://www.ncbi.nlm.nih.gov/pubmed/21406165> |
| HTR2C | Anxiety | <http://www.ncbi.nlm.nih.gov/pubmed/17240844> |
| HTR2C | Schizophrenia | <http://www.ncbi.nlm.nih.gov/pubmed/15717293> |
| HTR2C | weight_gain | <http://www.ncbi.nlm.nih.gov/pubmed/17702092> |
| IL6 | thrombocythemia | <http://www.ncbi.nlm.nih.gov/pubmed/11675343> |
| LPL | atherosclerotic_vascular_disease | <http://www.ncbi.nlm.nih.gov/pubmed/10580081> |
| LPL | Cachexia | <http://www.ncbi.nlm.nih.gov/pubmed/1638523> |
| LPL | coronary_artery_disease | <http://www.ncbi.nlm.nih.gov/pubmed/11015339> |
| LPL | disorders_of_lipid_metabolism | <http://www.ncbi.nlm.nih.gov/pubmed/17284417> |
| LPL | hypertriglyceridemia | <http://www.ncbi.nlm.nih.gov/pubmed/17560523> |
| LPL | Pancreatitis | <http://www.ncbi.nlm.nih.gov/pubmed/18936103> |
| MAOA | major_depressive_disorder | <http://www.ncbi.nlm.nih.gov/pubmed/18337637> |
| POMC | hyperpigmentation | <http://www.ncbi.nlm.nih.gov/pubmed/2831264> |
| PPARA | weight_gain | <http://www.ncbi.nlm.nih.gov/pubmed/12676649> |
| PPARG | Lipodystrophy | <http://www.ncbi.nlm.nih.gov/pubmed/17766367> |
| PPARG | Obesity | <http://www.ncbi.nlm.nih.gov/pubmed/17898990> |
| PPARG | type_2_diabetes | <http://www.ncbi.nlm.nih.gov/pubmed/18316027> |
| SERPINE1 | Thrombophilia | <http://www.ncbi.nlm.nih.gov/pubmed/14653439> |
| SERPINE1 | vascular_disease | <http://www.ncbi.nlm.nih.gov/pubmed/19132220> |

Sibutramine is a centrally-acting serotonin-norepinephrine reuptake inhibitor, structurally related to amphetamines. It was prescribed as an anorexiant before its withdrawal from the market due to severe adverse effects such as heart attacks and stroke. Some of the other common side effects include anxiety, nausea, vomiting, diarrhoea, constipation, and headache. Using comprehensive map, it is possible to predict the action of sibutramine on targets such as SLC6A3 and SLC6A4. Due to non specific binding of sibutramine to HTR2A, HTR2C, DRD2, COMT and MAOA (I Fig ), the side-effects such as nausea, vomiting, depression & anxiety can be explained (I Table). Thus, the comprehensive map can help in understanding the underlying mechanism of the drug-targets interactions. The results of docking of sibutramine with the molecules of comprehensive map are available in Supplementary Table 22 (website).


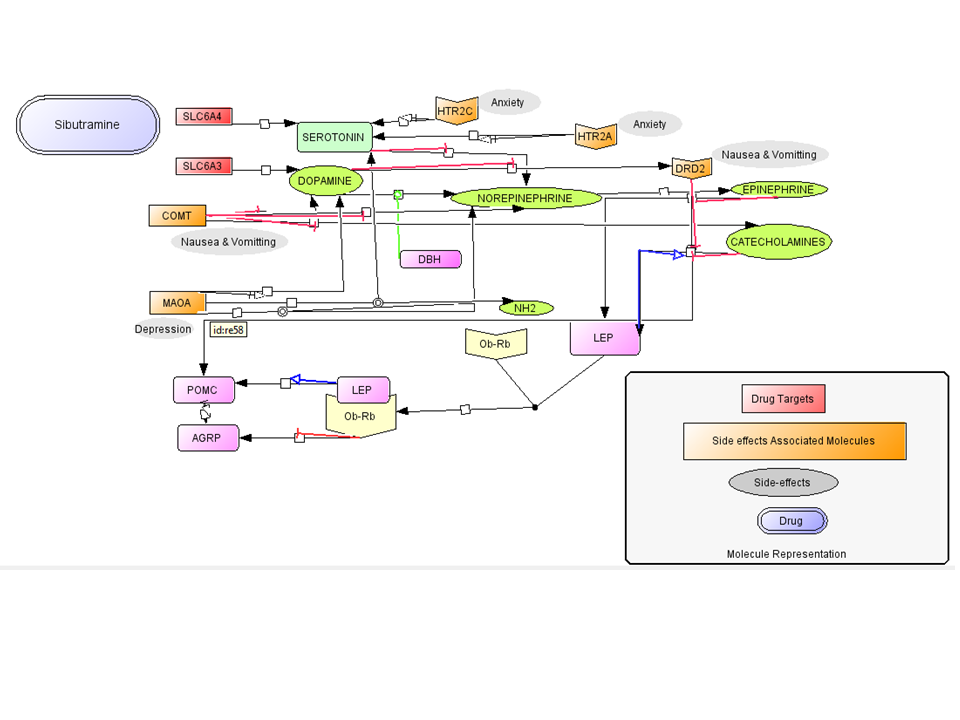


**I Fig.** A schematic diagram showing side effects of sibutramine due to non specific binding to off targets such as HTR2C & HTR2A (causing anxiety) as well as DRD2 & COMT (nausea and vomiting).

**References**

1. Hopkins *A.* Network pharmacology: the next paradigm in drug discovery. *Nature Chemical Biology. 2008; 4*: 682-690*.*
2. Campillos M, Kuhn M, Gavin A, Jensen L, Bork P. Drug Target Identification Using Side-Effect Similarity.Science AAAS. 2008; 321: 263-266.
3. Wallach I, Jaitly N, Lilien R. A Structure-Based Approach for Mapping Adverse Drug Reactions to the Perturbation of Underlying Biological Pathways. Plos One. 2010; 5: 1-11.

**F File**

**Comparison of Networks**

Kitano et al [1] built a network of metabolic syndrome for adipocyte, hepatocyte, skeletal muscle and pancreatic beta cell in 2004. We compared molecules of ON with this dataset (See J Table).

**J Table.** Comparison of molecules in Comprehensive Map with Metabolic Syndrome Molecular Map [1].

|  | **Nodes** | **Edges** | **Intersection** | **Difference** | **% Overlap** |
| --- | --- | --- | --- | --- | --- |
|  |  |  |  |  |  |
| **Comprehensive map (A)** | 803 | 976 | A∩B:108 | A-B:695 | 9.4 |
|  |  |  |  | B-A:232 |  |
|  |  |  |  |  |  |
| **Adipocyte ( B)** | 340 | 250 | A∩C:93 | A-C:710 | 8.05 |
|  |  |  |  | C-A:258 |  |
| **Hepatocyte (C)** | 351 | 272 |  |  |  |
|  |  |  | A∩D:109 | A-D:694 | 9.1 |
| **Beta cell (D)** | 382 | 272 |  | D-A:273 |  |
|  |  |  |  |  |  |
| **Skeletal muscle (E)** | 377 | 282 | A∩E:100 | A-E:703 | 8.4 |
|  |  |  |  | E-A:277 |  |

Logsdon et al [2] constructed mouse gene network using Bayes’ theorem. Out of 18 molecules reported by them, we found 3 molecules in our network constructed through deep curation system whereas text mining system reported match with 9 molecules of Logsdon work. (S10 Table at website).

We searched molecules reported by Logsdon et al [2] in obesity abstracts using perl scripts (text mining approach) to find the frequency. These molecules were also checked in the list of molecules generated from DC approach for their presence.

**K Table.** Comparison of molecules from Comprehensive Map with [2] Mouse gene network. See this table at website.

**References**

## Kitano H, Funahashi A, Matsuoka Y, kanae O. Using process diagrams for the graphical representation of biological networks. ***Nature Biotechnology. 2005; 23***:961– 966.

## Logsdon B, Hoffman G, Mezey J. Mouse obesity network reconstruction with a variational Bayes algorithm to employ aggressive false positive control. BMC Bioinformatics. 2012; 13:53:1471-2105.

**G File**

**Text-Mining (TM) Methodology & Tutorial**

The network was generated using steps described below.

1. All the text (.txt) and executable files (.exe) are available in Supplementary Folder 3 at “http://tinyurl.com/nzajxwe” for download.
2. The detailed tutorial is uploaded on website and only the essential steps are listed below.

**Step 1: Information Retrieval- Download abstracts from Pubmed using RefNavigator:**


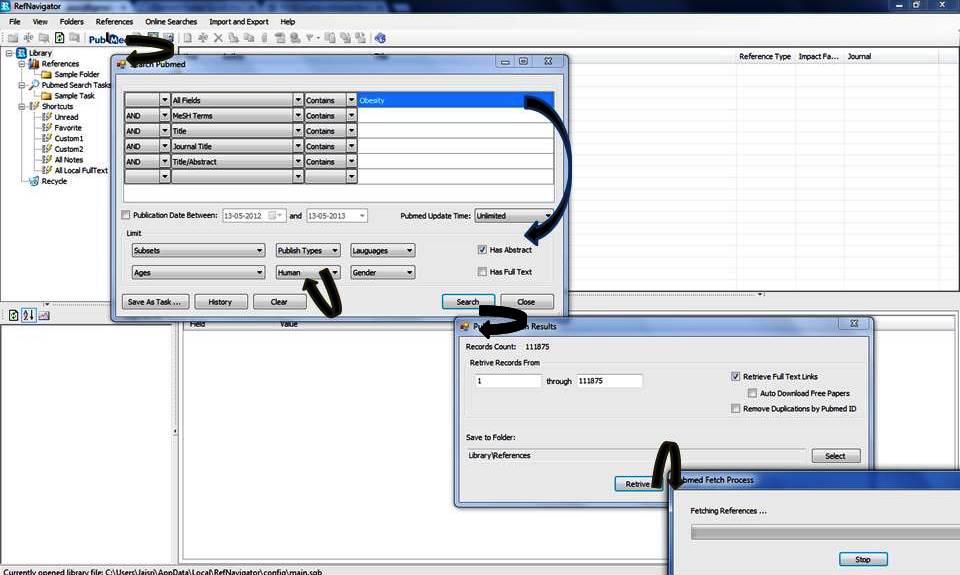


**J Fig.:** shows RefNavigator and its process.

**Step 2**:

1. **Pre-processing of the abstract file i.e. removing redundancy, blank space etc.**
2. **Knowledge Discovery Operations:**
3. **Compilation of gene list.**
4. **Identification of genes implicated in obesity.**
5. **Processing of the genes screened in step (ii).**
6. **Finding the co-occurrence data.**
7. **Using NLP for data processing:**

- **Creation of dictionaries for verbs denoting interactions.**
- **Screening of results with “Subject+Verb+Object” syntax.**
- **Screening of results with “Subject+Tense+Verb+Object” syntax.**
- **Screening of results with negations.**

1. **Graphical representation**

**Step 3: Visualization of the network**


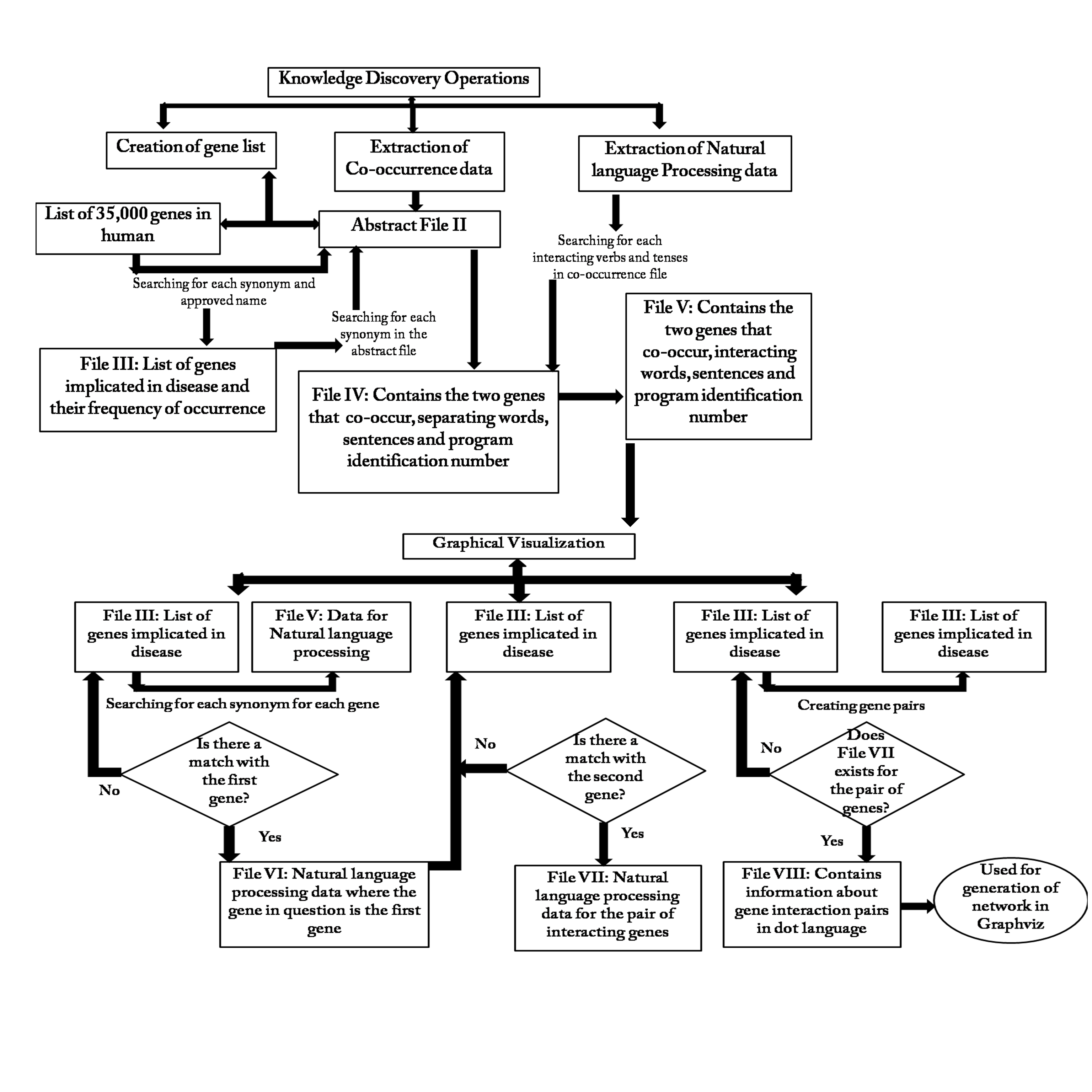


**K Fig.** shows the flow chart of various steps used in text-mining approach.

**L Table.** List of Dictionaries.

| **Positive Verbs** | **Negative Verbs** | **Neutral Verbs** |
| --- | --- | --- |
| Accelerate | Inhibit | Associate |
| Augment | Abolish | co-precipitate |
| Enhance | Block | Involve |
| Induce | down-regulate | Cause |
| Stimulate | Prevent | Relate |
| Require | Deactivate | Affect |
| up-regulate | Decrease | Contribute |
| Trigger | Diminish | Correlate |
| Bind | Control | Respond |
| Bound | Suppress | Result |
| Activate | Reduce | Confer |
| Activation |  | co-operate |
| Increase |  | Found |
| Express |  | Exert |
| Potentiate |  | Elicit |
| Phosphorylated |  | Influence |
|  |  | Predict |
|  |  | Alter |
|  |  | Develop |
|  |  | Link |
|  |  | Observe |
|  |  | Modify |
|  |  | Mediate |
|  |  | Modulate |

**M Table.** List of Tenses with examples.

| **Tense** | **Sentence** |
| --- | --- |
| Simple Present | A letter is written by Rita |
| Simple Past | A letter was written by Rita |
| Present Perfect | A letter has been written by Rita |
| Future I | A letter will be written by Rita |
| Hilfverben | A letter can be written by Rita |
| Present Progressive | A letter is being written by Rita |
| Past Progressive | A letter was being written by Rita |
| Past Perfect | A letter had been written by Rita |
| Future II | A letter will have been written by Rita |
| Conditional I | A letter would be written by Rita |
| Conditional II | A letter would have been written by Rita |

**N Table.** List of Negations.

| **Class I : To be used before the interaction verb** | |
| --- | --- |
| Tense | Form of Negation |
| Present Simple | do not ; does not |
| Past Simple | did not |
| Present Progressive | is not; are not |
| Past Progressive | were not; was not |
| Present Perfect | has not; have not |
| Present Perfect Progressive | has not been; have not been |
| Past Perfect | had not |
| Past Perfect Progressive | had not been |
| Future Simple | will not |
| Future Perfect | will not have |
| Conditional | would not |
| Conditional Perfect | would not have |
| Modals | cannot; should not |
| **Class II: To be used before the object gene** | |
| but not to |  |
| Whereas |  |
| While |  |
| but not |  |
| **Class III: To be used before the subject gene** | |
| independently of |  |
| inability of |  |

(A)**
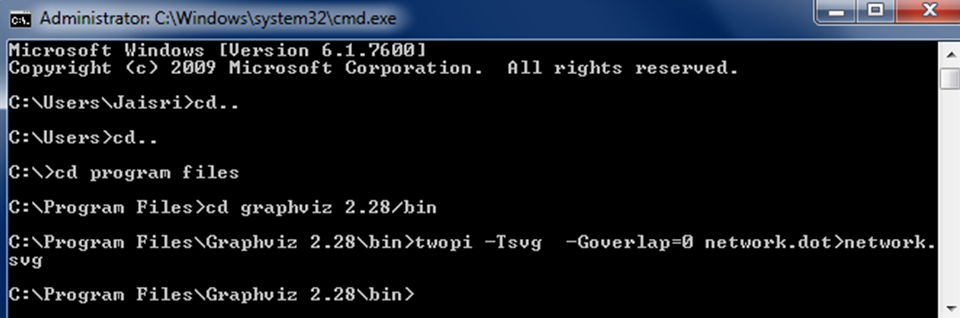
(B)
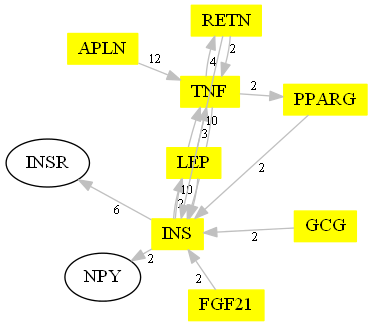
**

**L Fig.** shows (A) the command-line prompt and command for generating the network and (B) the network “*Network.svg”* generated with Graphviz.

**H File**

**Details of Annotation Process**

First, we constituted two teams of curators. Each team read the abstracts/full length papers independently & assigns each gene as an example associated with obesity. In case of disagreement, a third team headed by senior curator (expert) read the abstracts/full length papers again to resolve the issue. Additional evidences (abstracts/papers) were also used to resolve the conflict. We read the full length papers in deep curation approach, whereas for text mining system, we used perl scripts for screening of abstracts. Subsequently, results were screened manually to check the accuracy of text mining system.

**Determining True Positives and False Positives**

Researchers have used several approaches to link genes with complex traits such as obesity. Primarily, linkage analysis and association studies have been used to find the variants that affect obesity. In addition, animal models also provide list of candidates genes through linkage studies, expression profiling, and transgenic strains. The techniques such as expression analysis and protein interaction studies also identify candidate genes for obesity.    Given the wide variety of experimental techniques, we grouped these studies (evidences) into various categories and provided a numerical code to each of them (See B Table in S2 File). We also label each gene on the basis of the evidence along with evidence code for better data management.

A gene is defined as true positive example, when we have high confidence evidence to link a gene with a disease. For example, Leptin (Lep) deficiency [1] is linked with intractable form of obesity (Uniprot Id - P41159; OMIM ID- 614962). The false positives are those gene examples which matched common English words used in sentences, abbreviations of organizations, and author names. They also include those genes which occurred in obesity abstracts but rejected during manual screening due to lack of clear evidence (See B Table in S2 File).

We searched set of 35,959 genes in a sample set of approximately 96,219 abstracts. Through text-mining, we found 4,274 genes as first round of ‘hits’ (See Methods and Supplementary data). Since text mining systems are known to produce large number of false hits, therefore we screened these hits manually in reported abstracts and removed gene names matching with common English words; abbreviations and methodology terms using various types of filters (See examples provided on our website “<http://tinyurl.com/d74r9xy>”). Out of 4,274 hits, we label 1,268 genes as true positive hits and 3,005 false positive hits (See C Table in S2 File). These 1,268 gene examples were supported by at least one study with clear experimental evidence (See S3 File).

The goodness of annotation function can be estimated by standard measures used in text mining known as precision and recall. The precision is defined number of correct predictions divided by total number of predictions. In our case, it was found to be 30 % suggesting large number false positives generated during text mining based searches.

**Reference**

[1] Strobel A, Issad T, Camoin L, Ozata M & Strosberg A.D. A leptin missense mutation associated with hypogonadism and morbid obesity. Nature Genetics.1998; 18:213 – 215.

**I File**

**Composite Metric Score for Literature Mining**

We assume that two terms are highly related if they appear frequently together in the text. We define a parameter as W(C,g) to find association strength between a gene (g) and a clinical condition (C). Thus, W(C,g)  measures occurrences of keyword ‘g’ in abstracts relevant to the clinical condition (C). Next, we define a corpus of literature data *C_i_* as set of all abstracts: 96,219 abstracts (obesity abstracts till December 2012). Thereafter, we search a gene (leptin) and its synonyms in this dataset. We found leptin in 7159 abstracts out of 96,219 abstracts (7.4% of total dataset) of *C_i_*. Similarly, we measure the frequency of all the genes including major hubs (important genes) in context of obesity. We also created control groups for comparison: lesser-studied (reported) group (See manuscript for details), group of genes unrelated to obesity, and randomly selected genes. Next, we searched member of these groups in *C_i_* . We observed that frequencies of hubs are significantly higher than that of genes from control groups (P <0.05) (O Table). We find that chance of finding a gene or molecule unrelated to obesity (e.g. growth associated protein 43, GAP-43) is comparatively very less (0.002 %).

**O Table. Frequency of occurrences of molecules in obesity abstracts. Four categories are listed: (1) implicated in obesity (Hub Genes), (2) lesser-studied (reported) genes implicated in obesity, (3) randomly selected genes from HUGO database and (4) disease genes not related to obesity.**

|  | **HUB Genes** | **Count** | **Frequency** | **Randomly Selected genes** | **Count** | **Frequency** | **Diseases Genes not related to obesity** | **Count** | **Frequency** | **Lesser studied Genes** | **Count** | **Frequency** |
| --- | --- | --- | --- | --- | --- | --- | --- | --- | --- | --- | --- | --- |
| **1** | **PPAR Gamma/PPARG** | **1550** | **0.016109** | **PRKAB1** | **3403** | **0.035367** | **FANCI** | **2** | **0.0000207** | **WT1** | **19** | **0.000197** |
| **2** | **Insulin** | **23165** | **0.240753** | **UBE2NP1** | **0** | **0** | **ARFL1** | **0** | **0** | **DRD3** | **2** | **0.000020** |
| **3** | **Leptin** | **7159** | **0.074403** | **SYCP1** | **0** | **0** | **RAD2** | **13** | **0.0001351** | **FGFR3** | **10** | **0.00010** |
| **4** | **TNF alpha** | **2793** | **0.029027** | **ICAM1** | **291** | **0.003024** | **RIC8A** | **0** | **0** | **CDH2** | **2** | **0.0000207** |
| **5** | **GLUT4** | **383** | **0.003980** | **DGAT1** | **58** | **0.000602** | **RPL15P12** | **0** | **0** | **CYP2D6** | **2** | **0.0000207** |
| **6** | **FABP4** | **155** | **0.001611** | **BDMR** | **1** | **0.000010** | **TROAP** | **2** | **0.0000207** | **ALMS1** | **28** | **0.000291** |
| **7** | **PKA** | **5549** | **0.057671** | **COPZ2** | **0** | **0** | **AAGAB** | **1** | **0.000010** | **GABRG3** | **4** | **0.000041** |
| **8** | **PAI-1** | **12231** | **0.127116** | **BBS9** | **4** | **0.000041** | **GAP-43** | **3** | **0.000031** | **FMR1** | **160** | **0.001663** |
| **9** | **C/EBPalpha** | **120** | **0.001247** | **ZWILCH** | **0** | **0** | **SART1** | **0** | **0** | **PWCR1** | **16** | **0.0001663** |
| **10** | **C/EBPbeta** | **54** | **0.00056** | **TMEM176B/LR8** | **0** | **0** | **RPL15P12** | **0** | **0** | **BBS3** | **18** | **0.0001870** |

As an additional control, we also computed the frequency of occurrence of obesity related genes (i.e. hubs) in asthma abstracts (C_asthma ~_ 68,484 abstracts extracted for year 1960-2011). We observed that propensity to find hubs is significantly different (P<0.05) in asthma dataset. Likewise, we searched asthma candidate genes in asthma dataset as well as in obesity dataset and obtained similar results.

To check the role of paucity of literature data and other relevant factors on association strength, we used Google scholar to get relevant data since it provides highly cited papers as top hits along with other information. Here, we propose a composite metric to estimate the association of genes w.r.t. obesity by integrating information from different sources.

First, we searched Google scholar with key words “insulin gene and obesity” and found 1,190,000 hits.  Out of these hits, we select top 10 hits which include highly cited studies showing role of insulin gene in obesity.

A)   Manual Screening Score (M_s_): We read top 10 abstracts from Google scholar and if all 10 abstracts show positive role of insulin in obesity, we assign a score of 1. If 50 % of abstracts, show positive relation, a score of 0.5 is assigned. In case, all the 10 abstract shows no relation, we assign a score of 0.

B)    Frequency Score (F_s_): This score estimate the relative importance of a gene in a clinical condition based upon overall frequency of gene-disease co-occurrence in abstracts. For instance, term ‘insulin’ is present in 24.1% of 96,219 obesity abstracts dataset ( P Table); hence we assign a score of 24.1 to insulin using eq. 1.

……………… (**eq. 1)**

Y= count of gene (g) in condition (C) in pubmed abstracts.

X= Abstract count for condition (C).

C)    Citation Factor (C_f_): takes into account relative weightage of a study as well as journal reputation/impact factor by computing the average of citations received by top 10 studies. For example, top 10 papers for insulin gene’s role in obesity received average of 2570.1 citations (See Supplementary Folder 9 at website and P Table).

**P Table. Composite score distribution for genes labelled as hubs in obesity abstracts (till year 2015).**

| **GENE NAME** | **FREQUENCY (F_s_)** | **CITATION FACTOR (C_f_)** | **MANUAL SCREENING (M_s_)** | **COMPOSITE SCORE** |
| --- | --- | --- | --- | --- |
| **(Hubs)** |
| **PPARG** | **0.016** | **1046.1** | **0.8** | **13.3901** |
| **INSULIN** | **0.241** | **2570.1** | **0.8** | **495.515** |
| **LEPTIN** | **0.074** | **1549.5** | **0.7** | **80.2641** |
| **TNF alpha** | **0.029** | **893.6** | **0.9** | **23.323** |
| **GLUT4** | **0.004** | **1227.2** | **0.1** | **0.49088** |
| **FABP4** | **0.002** | **275.5** | **0.4** | **0.2204** |
| **PKA** | **0.058** | **747.8** | **0.2** | **8.6745** |
| **PAI-1** | **0.127** | **334.2** | **0.9** | **38.1991** |
| **C/EBPalpha** | **0.0012** | **697.7** | **0.1** | **0.083724** |
| **C/EBPbeta** | **0.0005** | **179.9** | **0.2** | **0.018** |

**Q Table. Composite score distribution for genes labelled as lesser studied in obesity abstracts (till year 2015).**

| **GENE NAME** | **FREQUENCY** | **MANUAL SCREENING (M_s_)** | **CITATION FACTOR (C_f_)** | **COMPOSITE SCORE** |
| --- | --- | --- | --- | --- |
|  | **(F_s_)** |  |  |  |
| **WT1** | **0.000197** | **0.60** | **304.30** | **0.03596** |
| **DRD3** | **0.000020** | **0.60** | **278.30** | **0.00334** |
| **FGFR3** | **0.000082** | **0.60** | **307.00** | **0.0151** |
| **CDH2** | **0.000021** | **0.80** | **147.00** | **0.00247** |
| **CYP2D6** | **0.000021** | **0.20** | **76.30** | **0.00032** |
| **ALMS1** | **0.000291** | **0.90** | **229.60** | **0.0601** |
| **GABRG3** | **0.000041** | **0.70** | **234.80** | **0.00674** |
| **FMR1** | **0.001663** | **0.40** | **203.50** | **0.13537** |
| **PWCR1** | **0.0001663** | **0.90** | **321.20** | **0.04807** |
| **BBS3** | **0.0001870** | **1.00** | **291.60** | **0.05453** |

Final Composite Score (C_s_) is a product of M_s_, F_s_, & C_f_.  We calculated that it is possible to determine the association strength of a given gene to a clinical condition using C_s_. The value of C_s_ranges from 0 to ∞ and the larger value correspond to better association.  We computed C_s_ for hub genes in obesity network and also computed C_s_ for control groups namely genes unrelated to obesity and randomly selected genes (See CS.xlsx at website). We observed that it is possible to discriminate between well studied genes and less reported genes using C**_s_** . Further, we can also measure association strength of a gene w.r.t. clinical condition using this metric (See M Fig., Q Table). We also used asthma as a clinical control to estimate the C_s_ of hubs of obesity network in asthma literature (R Table).

**R Table. Composite score distribution for genes labelled as hubs in obesity network in asthma related abstracts (till year 2015).**

| **GENE NAME** | **FREQUENCY (F_s_)** | **CITATION FACTOR (C_f_)** | **MANUAL SCREENING (M_s_)** | **COMPOSITE SCORE FOR OBESITY GENES IN ASTHMA ABSTRACTS** |
| --- | --- | --- | --- | --- |
| **PPARG** | **0.00082** | **384.4** | **0.1** | **0.03143** |
| **INSULIN** | **0.0046** | **347.8** | **0** | **0** |
| **LEPTIN** | **0.00105** | **137** | **0.1** | **0.144** |
| **TNF alpha** | **0.00042** | **303.6** | **0.2** | **0.0257** |
| **GLUT4** | **0.0000146** | **25.6** | **0** | **0** |
| **FABP4** | **0** | **76.3** | **0** | **0** |
| **PKA** | **0.00073** | **103.1** | **0** | **0** |
| **PAI-1** | **0.06893** | **96.4** | **0.2** | **1.3289** |
| **C/EBPalpha** | **0.00015** | **156.9** | **0** | **0** |
| **C/EBPbeta** | **0.000087611** | **103.7** | **0** | **0** |


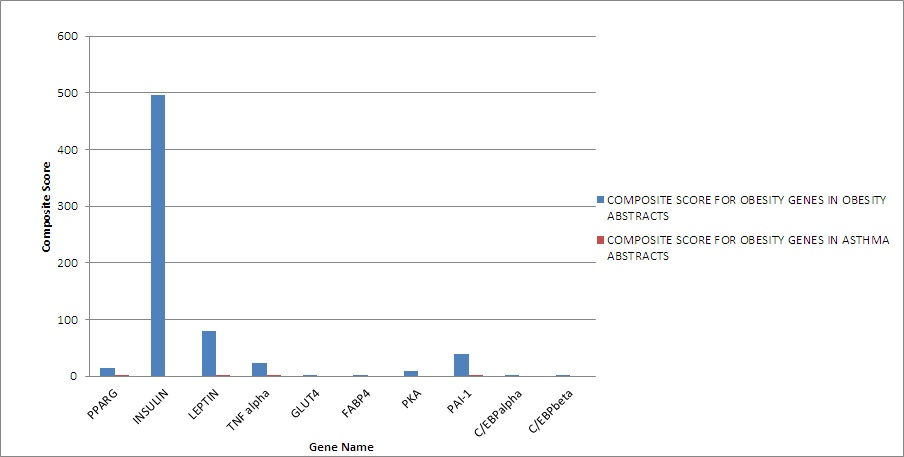


**M Fig. Distribution of composite metric score for various groups of genes in obesity literature and asthma literature.**

We conducted relevant statistical tests (i.e. T test and Mann–Whitney *U* test) using packages such as Minitab (www.mintab.com). We find that the composite scores for obesity hub genes in obesity related abstracts were significantly higher when compared with scores obtained in asthma related abstracts (Mann-Whitney Test p = 0.016; Fig M). In addition, composite scores of obesity hub genes were higher than that of any other sub groups or control groups in obesity abstracts.

**J File**

**Structure of Map**

The list of nodes included in the three indicated sub-network parts (top, central, bottom) is reported in Table 1. We evaluated the associations of these nodes with specific class of molecules using Bingo tool (See website).

**Representation of the map**

Genes and proteins are represented by standard notations, whereas interactions are categorized as positive, negative, neutral and catalysis. A positive regulation is defined for set of molecules, in which the molecule’s activity is stimulated by another molecule. In this context, an author frequently uses specific verbs such as stimulate, activate, induce, and enhance, up-regulate and increase. Negative regulation is the inhibition of the neighbouring interacting molecule which is evident by verbs like inhibit, down-regulate, decrease, prevent, suppress and reduce. Neutral regulation is the regulation between interacting molecules, the verbs includes: regulate, correlate, elicit, influence, associate, affect and contribute. Apart from these, there are some reactions where a molecule regulates the reaction between other molecules, i.e. catalysis. The edge representations include transcription, translation, association and dissociation using standard graphical notation (See Table 2). 

We used Cell Designer to create and visualize the comprehensive map. The Systems Biology Graphical Notation (SBGN) format is implemented by Cell Designer to distinguish the various types of molecules.

In this map, the yellow rectangular boxes with sharp ends are used to represent genes whereas green rectangular boxes with blunt ends are proteins. The green parallelogram represent RNA, ellipsoid with green shade shows a simple molecule and with grey shade represent the unknown molecule. The circular nodes in blue color represent ions and fluorescent blue colored oval shapes are used to depict a drug molecule. The complex of interacting molecules is shown as white boxes. The molecules implicated directly in obesity are assigned pink shade. The length of the edges in the comprehensive map was varied to make the graph layout optimally viewable (N Fig 1A and 1B). We also made a network in which area of node was proportional to degree of the node (O Fig.).


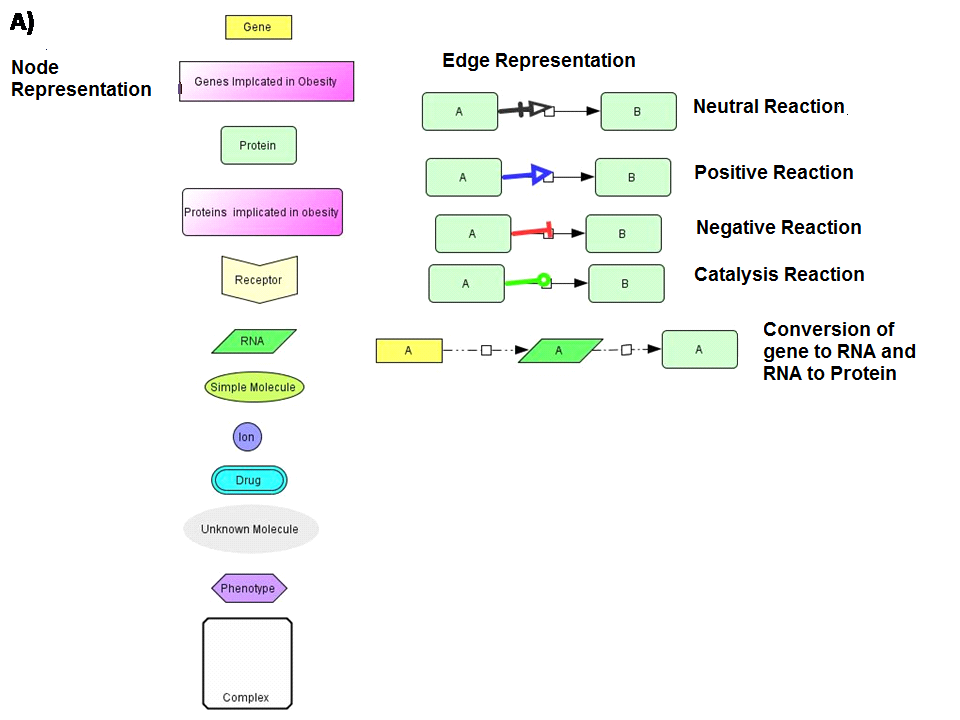


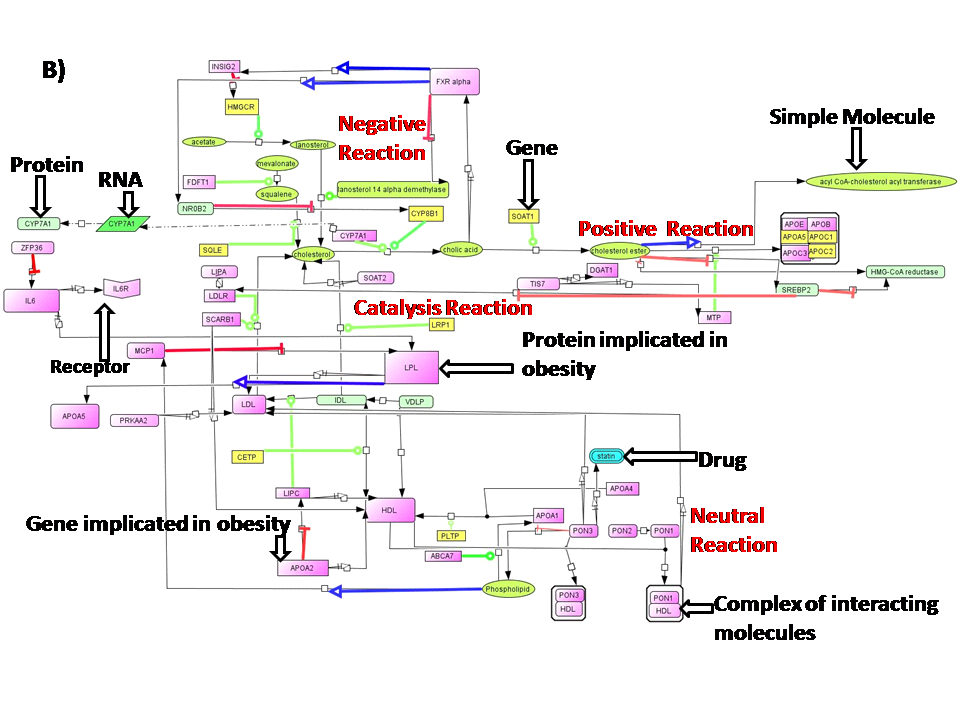


**N Fig.** (A) Represent the notations used to represent nodes and edges. (B) Shows a part of obesity network exhibiting notations used in Figure (A) as examples of genes, proteins, drugs, and interactions.


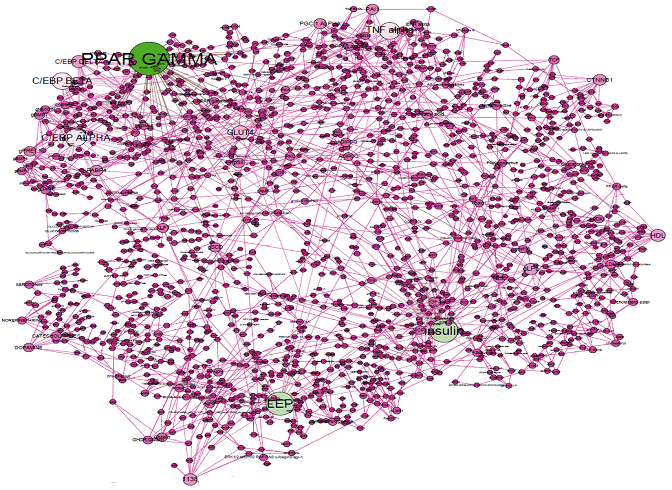


**O Fig.** Obesity network map was prepared using Gephi tool: The area of the circles (nodes) is proportional to the degree of the node.

**K File**

**Description of Docoviz**

Docoviz is a web-enabled perl based method to perform docking of drugs with large number of proteins.  It includes open source docking programs such as Autodock Vina [1]. User has an option to dock whole proteome (PDBs) or subset of proteome with one or multiple drugs. The program is made available on web with limited number of drugs and proteins. For large scale docking, user is encouraged to use executables provided over here (See website). The program has been tested on supercomputing facility at IIT Delhi (<http://www.scfbio-iitd.res.in/>) for large scale docking projects. End users are advised to use systems with at least 2 GB RAM on windows platform (XP, 7, 8, or 10).  For our research, we used supercomputing facility and we are in the process to include Apache hadoop framework (https://hadoop.apache.org/) to speed up the computational processes.

In the following section, we summarize the docoviz process (P Fig):

- First, we remove all the non-protein molecules from the pdb structure including water molecule, detergents, and ligands.
- Secondly, we convert protein pdb files to pdbqt format.
- Thirdly, the ligand with an extension *.mol file and *.mol2 file are converted into pdbqt format.
- Next, we identify the active site of the protein and generate a configuration file that contains the information of protein, ligand, protein active site coordinate and a grid size of 20 Aº (default parameter) to provide all possible accessible translation points surrounding any given cavity point.
- Finally, we use Autodock vina to dock a given ligand with set of processed protein molecules.
- Graphviz is also used to visualize drug interaction network based upon the binding energy value.


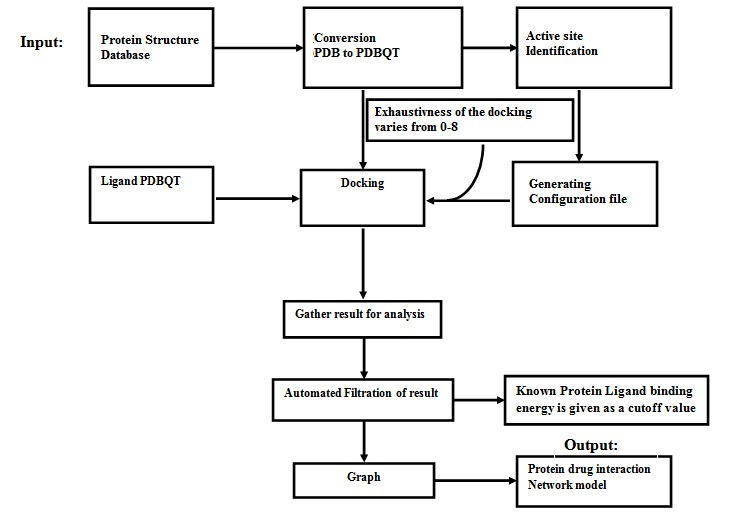


**P Fig.** A schematic diagram depicting work flow of Docoviz

**Applications:**

**Finding distribution of binding energies of drug target pairs using Docoviz:**

- **Preparation of target protein and small molecule library**

We downloaded proteins structures (humans) from RCSB protein databank as target proteins (<http://www.rcsb.org/pdb/home/home.do>) and small molecules from chemical databases such as Drugbank (<http://www.drugbank.ca/>) and ChEMBL (<https://www.ebi.ac.uk/chembl/compound>).

- **Training Dataset**

We constituted positive dataset for 23 drug molecule from drug bank along with respective protein target. In case, there are more than one crystal structure present in PDB, we selected only those protein with resolution better than 1.9 Aº. To create control dataset, we randomly selected 23 drug molecules having no connection with proteins of positive data set. Next, we identified the embedded ligand position in the x ray crystal structure using perl scripts. Finally, we docked known drugs with their respective targets. As a control same protein targets were used during docking experiments with randomly selected drugs (See S Table and T Table).

**S Table. Binding energies for docking of drugs in positive and control dataset.**

| **Protein Id** | **Protein name** | **Known Drug** | **Positive Dataset** (Kcal/ mol) | **Randomly selected Drug** | **Negative Dataset**  (Kcal/ mol) |
| --- | --- | --- | --- | --- | --- |
| 12CA | Carbonic anhydrase 2 | Topiramate | -9.9 | Pyridoxal Phosphate | -8.2 |
| 1A00 | Hemoglobin subunit alpha | Mefloquine | -9.4 | Tetrahydrofolic acid | -8 |
| 1A07 | Proto-oncogene tyrosine-protein kinase Src | Dasatinib | -8.5 | L-Histidine | -4.4 |
| 1A27 | Estradiol 17-beta-dehydrogenase 1 | 4-Androstenedione | -8.9 | Pyruvic acid | -2.7 |
| 1A2C | Prothrombin | Dabigatran etexilate | -6.3 | L-Phenylalanine | -4.6 |
| 1A2N | UDP-N-acetylglucosamine 1-carboxyvinyltransferase | Fosfomycin | -9 | Biotin | -5.2 |
| 1A31 | DNA topoisomerase 1 | Lucanthone | -6.8 | L-Lysine | -3.6 |
| 1A4G | Neuraminidase | Zanamivir | -5.2 | Vitamin C | -4.5 |
| 1A5H | Tissue-type plasminogen activator | Aminocaproic Acid | -3.7 | L-Aspartic Acid | -3.9 |
| 1A5K | Urease alpha subunit | Ecabet | -7.8 | L-Ornithine | -3.4 |
| 1A7X | FK506-binding protein 1A | Tacrolimus | -7 | L-Glutamine | -4.1 |
| 1AJ0 | Dihydropteroate synthase | Sulfacytine | -6.5 | L-Serine | -2.9 |
| 1AUE | Serine/threonine-protein kinase mTOR | Temsirolimus | -7.3 | Xanthophyll | -6.1 |
| 1AUI | Calcineurin subunit B isoform 2 | Cyclosporine | -8.1 | L-Cystine | -2.8 |
| 1AYP | Phospholipase A2, membrane associated | Indomethacin | -7.6 | N-Acetyl-D-glucosamine | -4.4 |
| 1AZM | Carbonic anhydrase 1 | Quinethazone | -6.5 | L-Glutamic Acid | -3.8 |
| 1B0P | Pyruvate-ferredoxin oxidoreductase | Nitazoxanide | -7.4 | Phosphatidylserine | -5.9 |
| 1B3O | Inosine-5'-monophosphate dehydrogenase 2 | Mycophenolate mofetil | -5.6 | Glycine | -2.1 |
| 1B5M | CYTOCHROME B5 | Alpha-Linolenic Acid | -6.7 | L-Leucine | -4.2 |
| 1B8Y | Matrix metalloproteinase-27 | Marimastat | -8.9 | L-Tryptophan | -7.2 |
| 1E2H | Thymidine kinase | Penciclovir | -6 | Chlorpromazine | -4.9 |
| 1E3G | Androgen receptor | Boldenone | -9.8 | Mibefradil | -1.6 |
| 1UWH | B-Raf proto-oncogene serine/threonine-protein kinase | Sorafenib | -10.8 | Diethylcarbamazine | -5.7 |

**T Table. Distribution of binding energies in positive and control dataset.**

| **T-test (Two Population) Paired test at significance level by default 0.05 [8/7/2014 16:24 "/Data1" (2456876)] Paired t-Test on Data1 col(A) and col(B):  Data Mean Variance N ------------------------------------------------------------ A -4.53043 2.99221 23 B -7.55217 2.88988 23 ------------------------------------------------------------ t = 7.4129 p = 2.04274E-7  At the 0.05 level, the two means are significantly different.** |
| --- |
|
|
|
|
|
|
|
|
|
|
|
|
|
|
|
|
|

- **Docoviz output**

Default parameters were used during docking and docking scores were computed for drug-target pairs for both positive and control dataset. We see significant difference in distributions of docking scores in positive and control datasets (Q Fig).


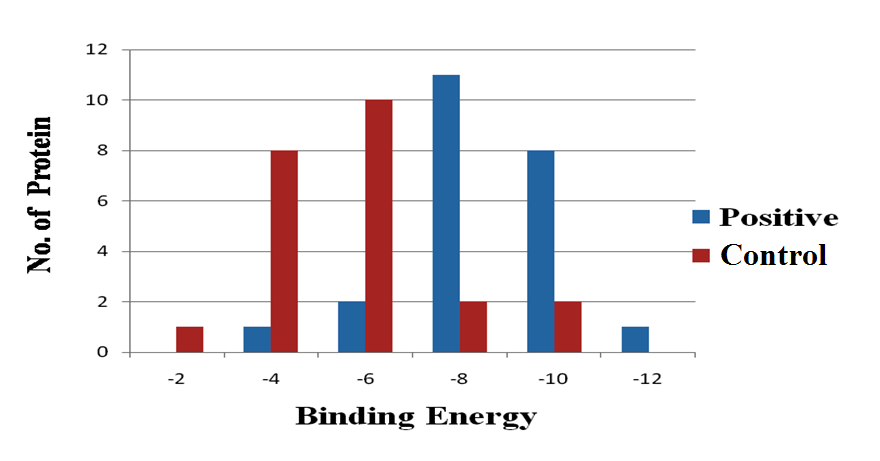


**Q Fig.** Distribution of Docking energies in Positive and Control Dataset (n=23; drug target pairs).

**Reference**

[1] Trott O, Olson AJ. AutoDock Vina: improving the speed and accuracy of docking with a new scoring function, efficient optimization and multithreading. J Comput Chem. 2010; 31:455–461.
