# Supplementary material for: Comprehensive Map of Molecules Implicated in Obesity
Source: PLoS One. 2016 Feb 17;11(2):e0146759. doi: 10.1371/journal.pone.0146759 (PMC4757102; doi:10.1371/journal.pone.0146759)
Supplement: S3 File — (DOCX) [file pone.0146759.s003.docx]

1. Website: <https://sites.google.com/site/obesitynetworkssupp/> for additional supplementary data.
2. <https://sites.google.com/site/obesityacomprehensivemap/>
3. <http://www.obesitynetwork.site88.net/>
4. **Mirror Sites:** https://figshare.com/articles/supplementary_Data/2060010
